# The AutGO Initiative: A Conceptual Framework for Developing Genetics‐Outcomes Research Hypotheses

**DOI:** 10.1002/aur.2331

**Published:** 2020-07-03

**Authors:** Zohreh Talebizadeh, Ayten Shah, AutGO Working Group

**Affiliations:** ^1^ Children's Mercy Hospital Kansas City MO USA; ^2^ University of Missouri‐Kansas City School of Medicine Kansas City MO USA

**Keywords:** autism, conceptual framework; genetics, outcomes, engagement, stakeholders, translational research

## Abstract

The increasing emphasis on translational approaches to complex neuropsychiatric and neurodevelopmental conditions research requires scientists from a broad range of disciplines to build dynamic collaborations when formulating hypotheses and framing study designs. The need to integrate the knowledge and perspectives not only from multiple scientific silos but also from the populations impacted by these conditions presents a significant challenge to researchers, particularly for a heterogeneous condition like autism. As one path toward addressing these challenges, we have previously introduced Autism Genetics Outcomes (AutGO), an initiative to support broad stakeholder partnerships and promote a new integrated concept called GO (i.e., research approaches that draw on both genetics and clinical outcomes perspectives). Herein, we developed a workflow for collecting stakeholders' feedback toward the development of a GO hypothesis. AutGO is an evolving initiative, and here we describe how its three essential components (conceptual framework, applicability, and implementation) have been developed. As a proof‐of‐concept, the AutGO team sought to demonstrate how a GO hypothesis could be developed using a semi‐structured literature review workflow. We also developed a prototype from published reports and formulated a GO hypothesis for autism. Rather than seeking community stakeholder input after a research project is conceptualized and designed, the developed conceptual framework demonstrates the feasibility of formulating scientific hypotheses by engaging stakeholders in retrospective semi‐structured literature reviews. The presented workflow, prototype, and discussed recommendations will bring awareness in the autism research community about the benefits of applying the GO approach in order to promote translational aspects in genetics research.

**Lay Summary:**

We used a community‐based engagement approach to develop AutGO (Autism Genetics Outcomes), an initiative to establish stakeholder partnerships and to promote research approaches (we refer to as GO) that draw on both genetics and clinical outcomes perspectives. Specifically, we developed a conceptual framework that includes a literature review process for developing GO hypotheses and stakeholder feedback collection protocol. Our work will bring awareness in the autism research community about the benefits of integrating patient perspectives in genetics research. ***Autism Res** 2020, 13: 1286–1299*. © 2020 The Authors. *Autism Research published by International Society for Autism Research* published by Wiley Periodicals LLC.

## Introduction

Extensive heterogeneity in the presentation of autism spectrum disorders (ASD) [Lord, Rutter, & Le Couteur, 1994] is a key challenge for understanding the genetic underpinnings of ASD, essential elements of translational research, and development of effective personalized treatments [Beversdorf and Missouri Autism Summit, 2016]. One approach to address this recognized challenge is to identify subtypes based on clinical and genetic information, for example, data archived in ASD repositories. The substantial investments made by the research community and participating patients in developing repositories indicate a high level of interest in combined assessments of genetic and clinical data. Patient contributions to such repositories have been mainly limited to providing biomaterials and clinical data, predefined by approved research protocols. While having this type of structured clinical data is essential for rigorous statistical analyses, it also poses a limitation of not capturing patient/parents observations and perspectives.

There are currently hundreds of candidate genes for autism. As such, the field of autism genetics has moved past the point of discovery into the realm of understanding how these genes contribute to abnormalities in neurodevelopment and eventual neural function. Understanding the mechanisms underlying variable expressivity of core behavioral symptoms, responsiveness to particular treatments, and nonbehavioral problems in autism is a critical step toward informing personalized approaches to treatment. To reach this tall goal in genetics, which is a key component of translational research, holistic approaches that incorporate perspectives of key stakeholders, including patients, need to be considered.

Patient advocates have long voiced concerns over the need to improve translational aspects of genetics research studies and to engage community members in the research process. Researchers have also noted a paucity of translational research in the genetics field. The concept of incorporating patient perspective in genetics research has been around for a long time and is well represented in the mental health literature. Since 1997, the focus of the CDC's Office of Public Health Genomics service has been on identifying, evaluating, and implementing evidence‐based genomics practices for prevention and control over the country's most prevailing diseases, through multiple federal and state‐level programs and agencies. Several reports have been published with an emphasis on the need to use the strategic collaborative engagement of all stakeholders across multiple sectors, including patients, family members, patient advocates, and community leaders, to move implementation science forward. While the majority of reports recognize the need to place the patient and family at the center of genomic medicine implementation, they only provide recommendations, but not actionable plans for building implementation science frameworks [Burton, Adams, Bunton, & Schroder‐Back, 2009; Lemke & Harris‐Wai, 2015; Roberts, Mensah, & Khoury, 2019].

A recent systematic review by Nunn, Tiller, Fransquet, and Lacaze [2019] evaluated public involvement in human genomics research projects worldwide. They found that only about 30% of the initiatives described public involvement in any capacity, and 2% involved public beyond data collection and at the level of co‐creation of the scientific process, so called “citizen science.” Clearly, there is a great need to increase public involvement in genetics research, and develop standardized methods of reporting their engagement; the impact of such involvement may be evaluated by assessing both qualitative and quantitative data [Nunn et al., 2019]. A series of guidelines have recently been developed by the National Institute of Mental Health workgroups (nimh.nih.gov), particularly, in relation to methodological aspects of genetics research studies, but they did not include any recommendation for incorporating patient perspectives in study design.

To determine how public engagement in genetics research design may be conducted, we had launched a unique initiative called Autism Genetics Outcomes (AutGO) [Talebizadeh, Shah et al., 2018]. The initiative is continuously evolving, with Phase‐I and Phase‐II completed recently. Previously we described Phase‐I activities [Talebizadeh, Shah et al., 2018], including the development of a conceptual connection between genetics research and patient‐centered outcomes research (for simplicity, hereafter we refer to “patient‐centered outcomes research” as “outcomes research”). To do so, we introduced a new integrated concept called GO (i.e., studies that utilize data and principles of both Genetics and Outcomes research) and identified barriers, facilitators, and needs for promoting this type of hybrid research approach. Furthermore, we reported lessons learned and suggested recommendations for the research community, including building GO multidisciplinary teams, raising awareness in the research community about the importance of conducting GO studies, developing effective educational protocols that include disease‐specific examples, and assessing existing resources [Talebizadeh, Shah et al., 2018]. These findings were taken into consideration when designing the present study.

This article describes Phase‐II activities: the development of a conceptual framework for building GO hypotheses for a specific condition (i.e., autism), including stakeholders' engagement process, educational materials, and prospective dissemination activities. Specifically, we formed a multidisciplinary AutGO team, developed a semi‐structured literature review process, built a workflow to collect stakeholders' perspectives/feedback, and developed a GO hypothesis for a topic related to autism. AutGO presents a conceptual framework to engage stakeholders in developing GO hypotheses that could be followed and further expanded by others.

## Methods

### *Obtaining IRB Approval*

Ethical approval for this study, which included forming an advisory board (AB), conducting focus groups, distributing surveys, and recruiting new members, was granted by the Office of Research Integrity at Children's Mercy Hospital. Informed consents, documenting their agreement to participate in the study, provide feedback, and contribute to the presentations and publications, were obtained from all study participants.

### *Establishing Partnership*

We formed a multidisciplinary AutGO team composed of 30 participants, including outcomes researchers, genetics researchers, clinicians, parents/patient representatives, as well as community and industry representatives, who became members of an AB. Eight of these individuals also served on our expert panel (EP), as shown in Figure S1. Eighteen members were involved from the inception of AutGO and took part in Phase‐I. Through their networks, 12 new members were recruited for Phase‐II. See Table S1 for more details on participants' background/expertise.

### *Participatory Methods*

We employed community‐based participatory research methods to engage participants and collect feedback through an iterative process. This strategy provided equal opportunity for a wide range of stakeholders to contribute throughout the study. Emails were the main method of communication for sharing study updates and materials. The educational workshop, webinars, and in‐person meetings were also used to inform and engage participants. A series of surveys were developed and distributed using the SurveyMonkey tool for obtaining participants' feedback to (a) assess the workshop, (b) conduct a semi‐structured literature review process for formulating a GO hypothesis, and (c) evaluate the overall satisfaction with the project. In brief, participants were asked to complete surveys utilizing the Likert scale and free‐text responses. More details on methods, research design, and data analysis are provided in Table S2, using the COREQ checklist [Tong, Sainsbury, & Craig, 2007].

## Results

### *AutGO Workshop*

To introduce the overall goals of the AutGO initiative, we held a workshop in Kansas City, Missouri in 2017 as a kickoff meeting for Phase‐II. In addition to live presentations, three video presentations were shown. To promote partnership among different stakeholders the following measures were implemented in organizing the workshop: (a) while the workshop was open to the public, select attendees were directly invited to ensure the diversity of the audience, (b) speakers were carefully selected to ensure the inclusion of a wide range of perspectives, including genetics and outcomes researchers, clinicians, and patient advocates, and (c) study objectives were shared with the speakers in advance, and a suggested outline was prepared for each presentation to keep the discussions focused on the workshop objectives. See the workshop website for more details (agenda, summary, video clips, and a copy of stakeholders' slide presentations along with the suggested outlines). We distributed a postworkshop evaluation survey to the attendees to obtain their feedback and to assess the level of satisfaction with the event. Forty‐eight out of 60 (80%) attendees responded, and overall, attendees expressed satisfaction with the workshop. The main suggestion was to devote more time to this type of event in the future. See Supplemental File for workshop attendees' demographic data (Table S3), survey responses (Table S4), and examples of attendees' direct quotes.

### *Introductory Webinar*

Due to the novel nature of the GO concept, an introductory webinar was prepared for AB members, to ensure their active engagement throughout the study. Two in‐person meetings with the local members were organized to discuss the project flow and obtain feedback. The local members' feedback was then used to prepare the educational webinar describing the overall goal of Phase‐II. The webinar contained a brief background and rationale for conducting GO projects. It was shared with all AutGO members via the following link: https://www.youtube.com/watch?v=QZ-3PoJUKrQ. Topics included: (a) overview of the relevant terminology, (b) study objectives, (c) lessons learned from Phase‐I, (d) expectations from Phase‐II, (e) examples of the potential GO projects, and (f) feedback obtained from the AutGO workshop attendees.

### *Semi‐Structured Literature Review Protocol Development*

The overall goal of Phase‐II was to develop a workflow for incorporating feedback from different stakeholders toward developing GO hypotheses. As previously described, GO projects utilize data and principles of both genetics and outcomes research [Talebizadeh, Shah et al., 2018]. As a result, such projects primarily focus on improving patient health outcomes, not identifying disease causality. In Phase‐II, we further expanded the GO concept description by defining the necessary aspects of such projects. In our definition, a GO project must have at least one component related to the following aspects: genetics, outcomes, and/or treatment.

**Figure 1:**
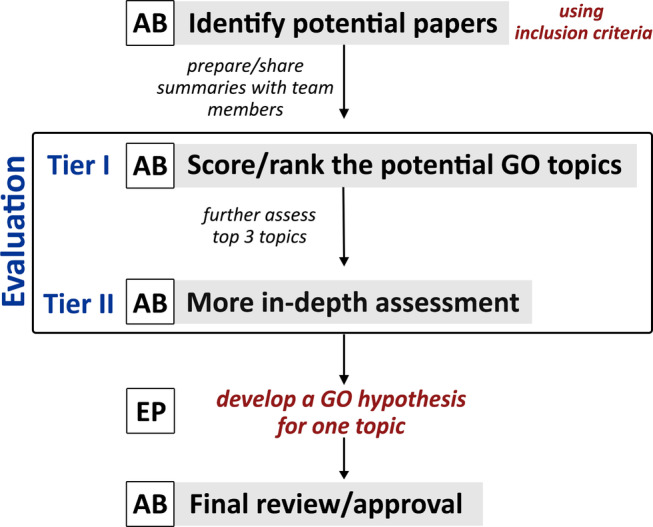
Semi‐structured literature review workflow. AB: advisory board; EP: Expert panel. The workflow was developed by incorporating participants' feedback obtained through other project activities: the AutGO workshop, in‐person meetings, and the educational webinar.

Once all members became informed about the overall goal by watching the educational webinar, the team worked together to develop a semi‐structured literature review pipeline for formulating a GO hypothesis. The pipeline included defining inclusion criteria (provided below) for the literature review, applying these criteria to select papers for team evaluation (participation in the papers nomination process was optional), and implementing a two‐step evaluation process (Tiers‐I and II) to obtain members' feedback, as illustrated in Figure 1.

### *Inclusion Criteria for Literature Review*

- Papers must include at least one of the following three elements:
  1. Patient health outcome(s),
  2. Genetic risk factor(s), and
  3. Combination of both.
- Papers must be related to one of the following conditions:
  1. Autism,
  2. Other neurodevelopmental disorders, and
  3. Comorbid conditions (i.e., symptoms that co‐occur with autism such as sleep problems, eating problems, etc.).

Using the inclusion criteria, AB members recommended papers for team evaluation. ZT and AS independently reviewed all suggested papers, with each paper being reviewed twice for accuracy. Together, they synthesized the information from the literature to narrow the papers down by themes to identify potential GO topics. A uniform structure was developed and used for summarizing each topic to facilitate the contribution of all members. Tier‐I topic summaries included: (a) *Main paper summary*: for each topic, one paper that had at least one GO aspect and could provide a foundation for developing a GO hypothesis, was briefly summarized, and (b) *Potential direction for developing a GO hypothesis*: a few additional points that could support each topic and direct future development of a GO hypothesis were gathered from the literature. Links to the papers were also provided. In Tier‐I assessment, Tier‐I topic summaries were reviewed by all members, and their feedback and topic rankings were collected through a Tier‐I survey.

Tier‐II topic summaries were prepared for the topics prioritized in Tier‐I, and included the following: (a) *Background*: A brief description about each topic was provided, (b) *Subtopics*: an additional literature search was conducted for each topic using the following search terms, while replacing “*topic*” with the respective terms provided in Figure S2: “gene & *topic,*” “outcomes & *topic,*” “autism & *topic,*” “treatment & *topic,*” “dietary supplementation & *topic,*” and “neurodevelopmental disorders & *topic*.” The information obtained from this targeted literature search was then used to identify a few relevant areas, or subtopics, which might be considered in developing a GO hypothesis. Subtopics were either directly associated with the topic of interest or related to one of the GO aspects. The GO aspects for each subtopic were described. A brief justification of why/how a subtopic could be related to building a GO hypothesis was also included (i.e., take‐home messages). (c) *A potential direction for developing a GO hypothesis*: An example of how the provided information could be used to develop a GO hypothesis was provided. (d) *Summary Table*: For convenience, subtopics and related GO aspects were also summarized in a table. (e) *Glossary*: A brief lay description of technical terms was provided to ensure that all AutGO team members, regardless of educational attainment, could readily evaluate the potential for the presented hypothesis to achieve clinical translation. In Tier‐II assessment, the Tier‐II topic summaries were evaluated by all members, and their feedback and topic rankings were collected through a Tier‐II survey. See Supplement File for an example of GO topic summaries.

### *Semi‐Structured Literature Review Protocol Implementation*

The developed literature review workflow was implemented by AutGO team members to formulate a GO hypothesis for one topic. Using the inclusion criteria, seven AB members recommended 60 papers, from which 10 potential GO topics were identified. These topics included self‐injury, depression, central auditory processing disorders, oxytocin, fever, mitochondrial dysfunction, metabolic problems, cognitive behavioral therapy, probiotics, and withdrawn behavior. In Tier‐I assessment, GO topic summaries for the 10 identified topics were distributed for AutGO members evaluation. The Tier‐I survey was used to collect feedback, based on which the following three topics received the highest ranking: mitochondrial dysfunction, depression, and probiotics. In Tier‐II assessment, more in‐depth summaries for these three topics were provided to AB members for further evaluation. Feedback was collected through the Tier‐II survey, based on which the topic of “depression in autism” was prioritized.

Next, eight EP members worked together to develop a GO hypothesis for the selected topic. Given that each member could have a different perspective, we divided the EP into EP‐I and EP‐II groups, each consisting of four members, and used a stepwise process for hypothesis generation to make the task more robust and manageable. Specifically, EP‐I drafted an initial version of the hypothesis and EP‐II further refined its components and overall direction. The hypothesis formulated by the EP members was then finalized by getting input from the rest of the AutGO team members through a survey. Figure S2 summarizes the steps taken and the results obtained at each step. See Supplement File for more details on the GO topic summaries and the developed hypothesis for “depression in autism.”

The top priority topics were identified by calculating the mean of scores of all AutGO team members, regardless of the stakeholder categories they represented. However, to see if there were any differences in responses for different stakeholder categories, corresponding numbers were also evaluated separately per each category, as shown in Tables 1 and 2, for Tier‐I and II, respectively. In Tier‐I, some similarities and differences could be seen between the rankings for different stakeholder categories. For example, depression was one of the top three prioritized topics selected by five out of six stakeholder categories, and four topics were uniformly given the lowest ranking by all stakeholders.

**Table 1:** Tier‐I Assessment Scores.

Topics/questions		Stakeholder category^a^						AutGO Team (*n* = 30)
		Researchers			Clinicians (*N* = 13)	Parents (*N* = 7)	Others (*N* = 10)	
		Genetics (*N* = 10)	Outcomes (*N* = 7)	Autism (*N* = 14)				
Please indicate to what extent you disagree or agree with the following statements with “0” indicating “Strongly disagree” and “10” indicating “Strongly agree”	1a. The summaries of the topics were provided in an easy to understand manner	9.7 (0.7)	9.3 (1.3)	9.4 (1.0)	9.0 (1.1)	9.4 (0.5)	9.5 (0.8)	9.3 (1.0)
	1b. The provided summaries represent a wide range of topics (i.e., have enough variability for consideration)	9.4 (0.8)	9.6 (0.5)	9.4 (0.9)	9.1 (1.0)	9.7 (0.5)	9.2 (1.0)	9.3 (0.9)
Overall, how satisfied or dissatisfied are you with the structure used for the topic summaries, which include the following: A brief (nontechnical) summary of the main paper. A suggested potential direction for a GO hypothesis. Links to the main and supporting papers. Glossary (with “1” indicating “Very dissatisfied” and “5” indicating “Very satisfied”)		5.0 (0.0)	4.9 (0.4)	4.8 (0.4)	4.7 (0.5)	5.0 (0.0)	4.9 (0.3)	4.8 (0.4)
Only three out of 10 topics will be selected for the next round of evaluation; please indicate to what extent you would recommend each topic with “0” indicating “Would not recommend” and “10” indicating “Would highly recommend”	Topic 1: Self‐injury and autism	7.8 (1.9)	7.9 (3.8)	7.4 (2.6)	5.5 (3.6)	7.9 (2.6)	6.6 (3.7)	6.7 (3.0)
	Topic 2: Depression and autism	7.1 (3.2)	8.9 (1.5)	7.6 (2.6)	6.6 (2.9)	8.3 (1.5)	7.7 (2.1)	7.4 (2.4)
	Topic 3: Central auditory processing disorders and autism	6.5 (3.4)	6.7 (2.6)	6.0 (2.8)	4.5 (2.0)	7.0 (2.6)	6.3 (2.3)	5.7 (2.5)
	Topic 4: Oxytocin treatment and autism	7.6 (2.5)	5.7 (2.1)	6.9 (2.5)	6.0 (2.6)	8.4 (1.3)	5.7 (2.5)	6.7 (2.4)
	Topic 5: Fever and autism	7.4 (2.4)	7.0 (2.8)	5.5 (3.0)	3.9 (2.7)	5.9 (1.9)	6.8 (2.4)	5.6 (2.8)
	Topic 6: Mitochondrial dysfunction and autism	6.6 (2.5)	7.0 (1.5)	5.9 (2.7)	5.7 (2.7)	7.3 (2.3)	6.8 (1.8)	6.4 (2.4)
	Topic 7: Metabolic problems and autism	7.5 (2.0)	7.3 (2.4)	7.2 (2.6)	6.4 (3.0)	6.9 (3.1)	7.3 (2.5)	7.2 (2.6)
	Topic 8: Cognitive behavioral therapy and autism	7.3 (2.2)	8.0 (1.6)	6.9 (2.3)	7.4 (2.6)	6.6 (2.4)	7.6 (1.8)	7.1 (2.4)
	Topic 9: Probiotics and autism	6.8 (2.7)	8.1 (1.9)	6.5 (2.3)	7.0 (1.5)	8.0 (1.4)	8.3 (2.5)	7.4 (2.1)
	Topic 10: Withdrawn behavior and autism	5.3 (1.8)	6.7 (1.6)	5.4 (2.6)	5.8 (3.3)	7.4 (1.6)	6.5 (1.6)	6.1 (2.4)

Mean and *SD* are provided for each stakeholder category as well as for the entire AutGO team.

a Some participants represented more than one stakeholder category.

**Table 2:** Tier‐II Assessment Scores.

Topics/questions		Stakeholder category^a^						AutGO Team (*n* = 30)
		Researchers			Clinicians (*N* = 13)	Parents (*N* = 7)	Others (*N* = 10)	
		Genetics (*N* = 10)	Outcomes (N =7)	Autism (N =14)				
Please indicate to what extent you disagree or agree with the following statement with “0” indicating “Strongly disagree” and “10” indicating “Strongly agree”	The detailed summaries of the three GO topics were provided in an easy to understand manner	9.7 (0.7)	9.4 (1.1)	9.2 (1.0)	8.9 (1.0)	9.7 (0.8)	9.6 (0.5)	9.3 (0.9)
Overall, how satisfied or dissatisfied are you with the structure of the GO topic summaries, which includes the following: Background, Subtopics, A potential direction for developing a GO hypothesis, Summary Table, and Glossary (with “1” indicating “Very dissatisfied” and “5” indicating “Very satisfied”)		4.8 (0.4)	4.9 (0.4)	4.9 (0.3)	4.8 (0.4)	5.0 (0.0)	4.9 (0.3)	4.8 (0.4)
Based on the provided detailed summaries, please indicate to what extent you would recommend each topic with “0” indicating “Would not recommend” and “10” indicating “Would highly recommend”	Depression and autism	8.5 (2.1)	9.3 (1.1)	8.6 (1.5)	8.1 (1.4)	9.4 (0.8)	9.4 (0.7)	8.6 (1.5)
	Metabolic problems and autism	7.4 (3.1)	7.1 (2.0)	7.4 (2.6)	7.2 (2.3)	8.9 (1.5)	7.7 (2.1)	7.6 (2.2)
	Probiotics and autism	6.0 (2.9)	6.7 (2.1)	5.8 (3.0)	5.9 (3.1)	7.9 (1.3)	6.7 (2.3)	6.6 (2.6)
Our goal is to develop a GO hypothesis for one topic only. Therefore, please rate these three topics on a scale of 1–3 with “1” indicating “Best preferred choice” and “3” indicating “Least preferred choice”	Depression and autism	1.4 (0.7)	1.0 (0.0)	1.4 (0.8)	1.7 (0.9)	1.7 (0.8)	1.3 (0.5)	1.5 (0.7)
	Metabolic problems and autism	2.0 (0.7)	2.3 (0.5)	1.9 (0.7)	1.8 (0.7)	1.6 (0.8)	2.1 (0.9)	2.0 (0.7)
	Probiotics and autism	2.6 (0.7)	2.7 (0.5)	2.7 (0.5)	2.5 (0.8)	2.7 (0.5)	2.6 (0.5)	2.5 (0.7)

Mean and *SD* are provided for each stakeholder category as well as for the entire AutGO team.

^a^ Some participants represented more than one stakeholder category.

**Figure 2:**
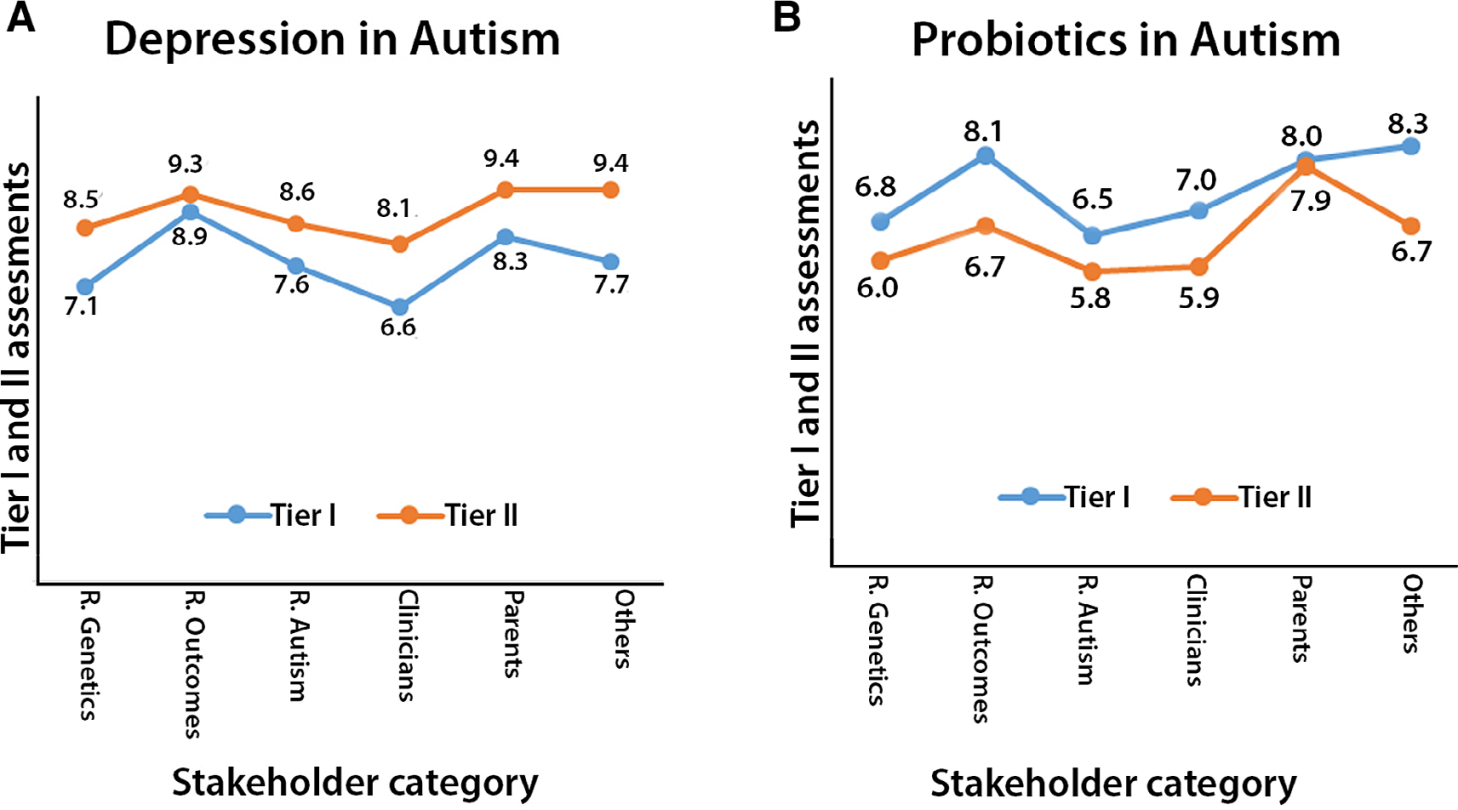
Graphs A and B show the scores given by each stakeholder group in Tier‐I and II assessments, for the topics of “depression in autism” and “probiotics in autism,” respectively. In both Tier assessments, team members were answering the same question: “Please indicate to what extent you would recommend each topic with “0” indicating “Would not recommend” and “10” indicating “Would highly recommend.” Uniform trends are seen when comparing the scores, even though, the two assessments were done independently by each participant. Of note, some members belonged to more than one stakeholder group and, in calculating averages, their scores were counted in all the groups that they have represented. Identifying why given topics may be perceived differently among stakeholder groups was beyond the scope of this study. Assessing such topics would require a larger sample size and different study designs. Stakeholder groups include Researchers (R) [R. Autism, R. Outcomes, and R. Genetics], Clinicians, Parents, and Others.

In Tier‐II assessment, two scoring systems were used to capture participants' perspectives about the top three topics. First, the same scoring as in Tier‐I was applied indicating the extent to which they would recommend each topic (i.e., a scale from 0 to 10 with “0” indicating “Would not recommend” and “10” indicating “Would highly recommend”). Second, members prioritized the topics to be chosen for the hypothesis development on a scale from 1 to 3, with “1” indicating “Best preferred choice” and “3” indicating “Least preferred choice.” Both scoring systems indicated that “depression in autism” was the top priority across all stakeholder groups. A comparison of Tier‐I and Tier‐II results for the topics of “depression in autism” and “probiotics in autism” (Fig. 2) showed a noticeable elevation in the scores (based on a scale from 0 to 10, with “0” indicating “Would not recommend” and “10” indicating “Would highly recommend” for “depression in autism,” and an overall decline for “probiotics in autism” across all stakeholder groups. Since the observed changes were relatively uniform across all groups, we assume that the additional educational materials provided in Tier‐II topic summaries could have contributed to the consistent changes across all groups.

### *Participant Retention*

Despite the complexity and novelty of the developed engagement protocol, we were able to maintain a 100% participant retention rate (i.e., all study participants remained active throughout the study process and completed the required surveys). Furthermore, all AutGO members expressed interest to be kept informed about future directions of the initiative and reported satisfaction with the way Phase‐II was conducted (see Supplemental File for selected comments).

### *Prototype: A Research Finding Exemplifying the GO Concept*

One effective way to illustrate the potential product of a conceptual framework is by prototype representation. Identifying a research example illustrating aspects of the GO concept may serve the prototyping purpose. As such, here we describe a successful story of the reported relationship between a variant in a gene called *MET* and gastrointestinal (GI) issues in autism, including how this discovery was made, the role of anecdotal observations, and the potential impact on patient health outcomes.

Parents have frequently raised concerns about GI issues in autism. Ellen Bolte, a mother of a boy with autism, first brought attention to a potential connection between her child's rapid developmental and behavioral regression and his GI symptoms. She had no formal medical or scientific background but started to study medical literature in a desperate attempt to find a solution that would ease her child's condition. In 1998, she published a perspective paper, describing her “gut bacteria theory” of autism [Bolte, 1998; Bolte, 2003]. Ellen outlined the possibility that in some cases of autism with regressive onset, behavior symptoms may have been caused by chronic neurotransmitter disruption in the brain due to the growth of neurotoxin‐producing bacteria in the intestinal tract after repeated antibiotic use. This and similar concerns raised by other parents contributed to bringing awareness in the research community about the potential co‐occurrence of GI issues in autism. In 2009, Campbell et al. identified the association between a *MET* variant in families with co‐occurring autism and GI conditions, by connecting phenotypic and genotypic data [Campbell et al., 2009]. In relation to the GO concept, it is important to highlight the following key points that have led to this discovery: (a) the dual function of *MET* (contributing to both GI functions and brain development) had already been documented in the scientific literature; (b) the awareness about GI issues in the ASD population, initially noted by parents, was confirmed by epidemiological studies; (c) taking into consideration *MET* functions and ASD phenotypic features (i.e., GI issues) shed light on the association of the *MET* variant in ASD subjects with GI issues. Thus, it is reasonable to conclude that the awareness about GI issues in this population, initially brought up by parents, contributed to the *MET* discovery.

**Figure 3:**
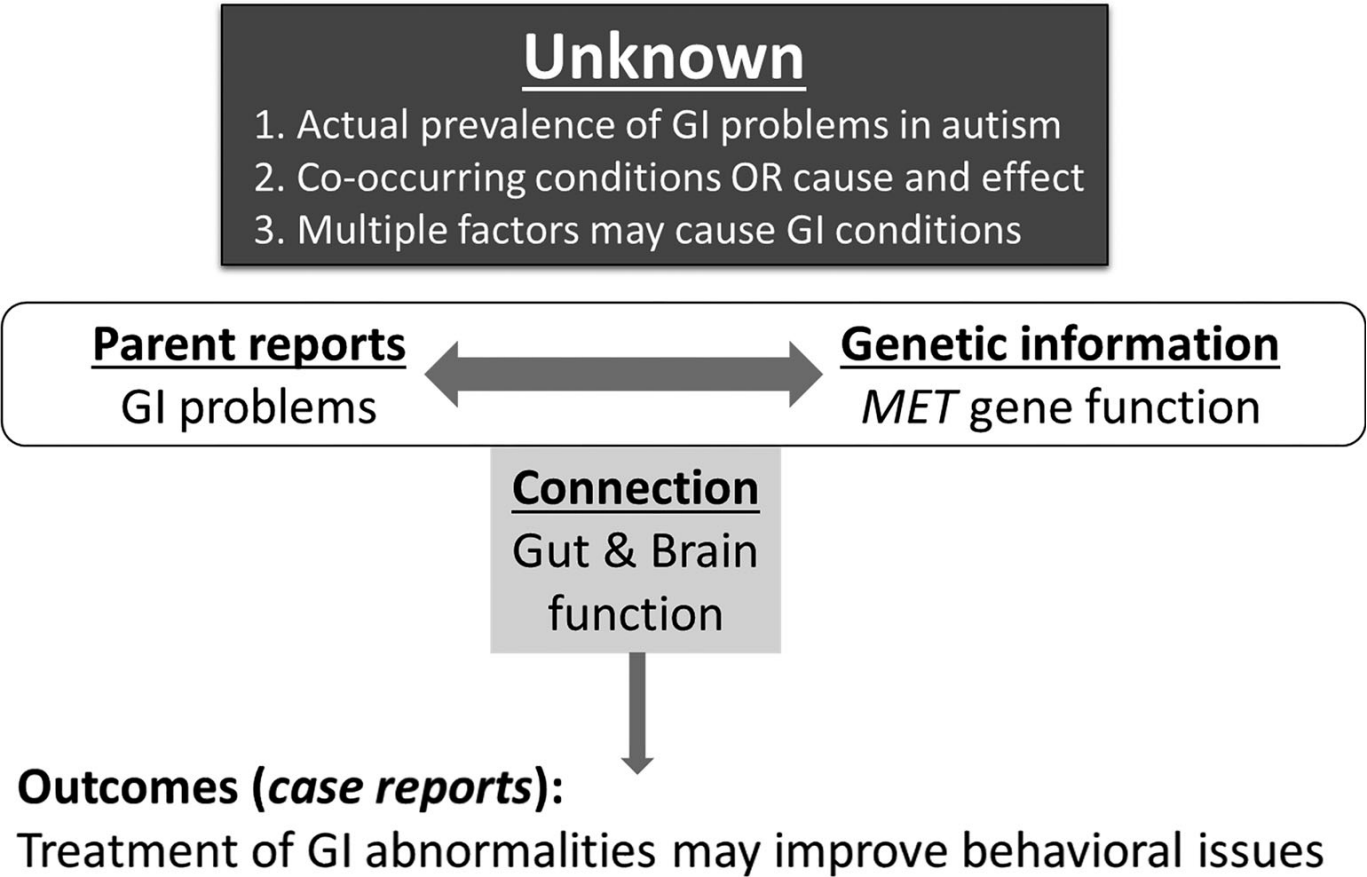
Prototype: A research finding exemplifying the GO concept. One example of a potential GO project is the successful story of the reported relationship between a variant in a gene called *MET* and GI issues in autism. Even though several factors in this model still remain unknown, taken together they led to a meaningful hypothesis and further highlighted the connection between gut and brain function [Campbell et al., 2009]. Connecting parent reports with genetic information contributed to this scientific discovery, positive outcomes of which have been reflected in some case reports.

As summarized in Figure 3, several factors still remain unclear in the *MET* success story. For example, we do not know: (a) what the actual prevalence of GI problems in autism is, (b) if these are co‐occurring conditions or have a cause and effect relationship, and (c) what the multiple genetic and nongenetic factors are that may contribute to GI conditions. Even though each of these items taken separately is not fully understood, taken together, they have led to a meaningful hypothesis and important findings. The take‐home message from this successful research discovery is that paying attention to the parent reports and connecting them with relevant scientific/biological knowledge has led to an important discovery and further highlighted the functional connection between gut and brain. Subsequently, more attention has been given to the importance of treating GI problems in individuals with autism, particularly for nonverbal cases. For example, some case reports showed that treating GI abnormalities in kids with autism might also improve their behavioral issues, and this is a meaningful outcome, for at least a subset of patients. Moreover, the first guidelines for healthcare providers on how to recognize [Buie et al., 2010 Report] and methodically evaluate GI issues in children with autism have been published [Buie et al., 2010 Recommendations]. More recently, a parent‐report screen for common GI problems in ASD has been developed to further facilitate early detection and health management in this patient population [Margolis et al., 2019]. This prototype offers an example of how initiatives like AutGO can help identify genetics research hypotheses that are more likely to translate from bench to bedside.

## Discussion

In addition to the phenotypic information collected for clinical and/or research level diagnosis, ASD subjects often present with other symptoms that are not part of core diagnostic features. For example, it has long been known that in addition to the well‐documented behavioral‐related issues defined by diagnostic criteria, parents also notice nonbehavioral problems in their autistic children (e.g., eating behaviors, sleep problems, etc.). Scientists often view these observations as anecdotal (i.e., informal personal testimony that do not qualify as scientific evidence). Thus, these informal reports are not usually considered in research studies. There are also clinical observations such as GI issues [Buie et al., 2010], neurological symptoms, and sensory‐motor deficits [Gillberg, 2003] that are not routinely incorporated in ASD genetic research protocols. However, informal evidence is potentially valuable for research purposes and should not be overlooked.

**Figure 4:**
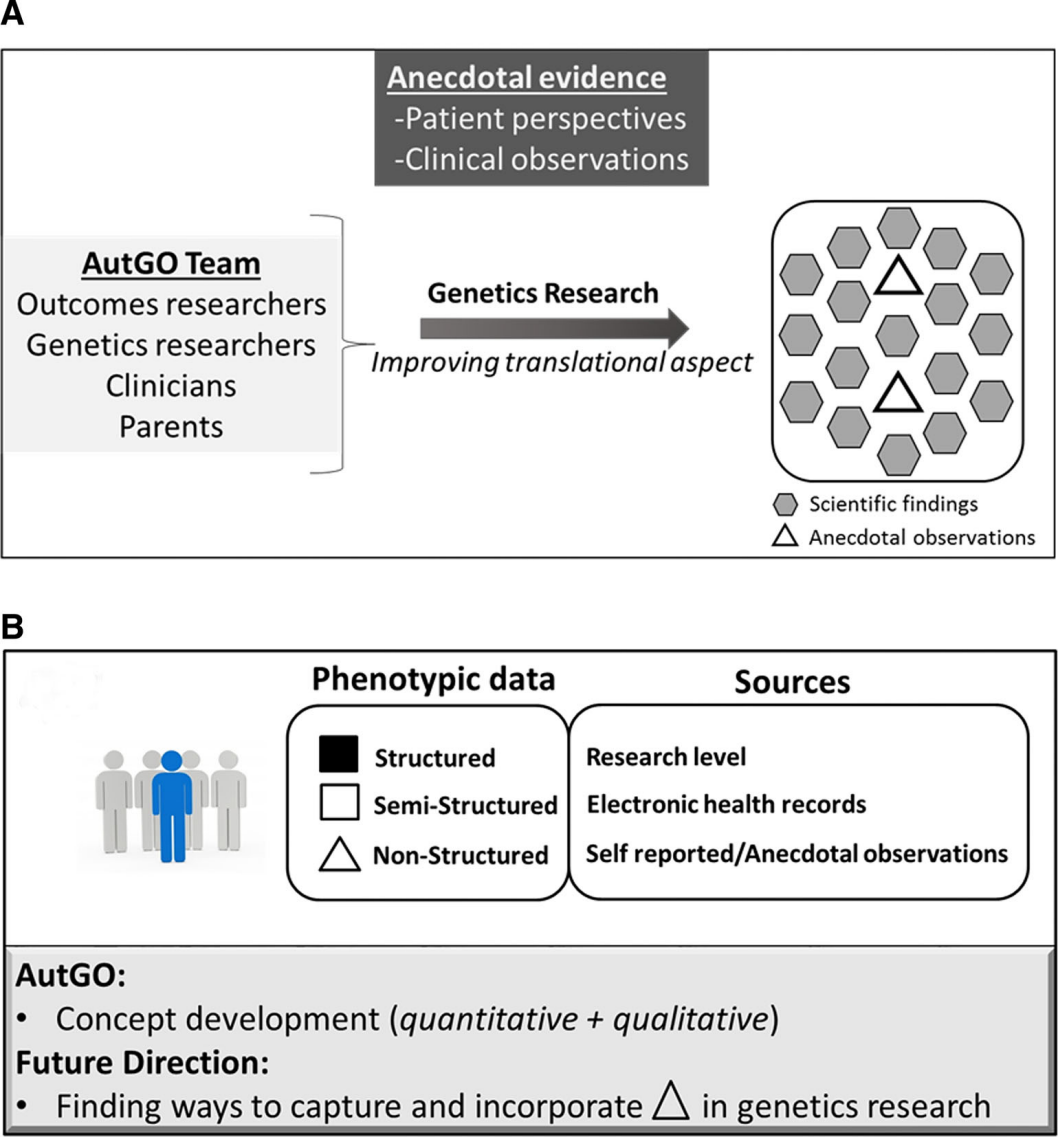
Schematic illustrating the potential value of incorporating anecdotal evidence in research. (**A**) One way to do so is to form multidisciplinary teams, such as AutGO, and incorporate stakeholders' perspectives in the research development process. (**B**) Phenotypic data representing patient profile may come from three different sources: research level, electronic health record, and self‐reported data, and be categorized as structured, semi‐structured, and nonstructured, respectively. The AutGO initiative provides a platform for the concept development and potential future directions.

Parent concerns are usually communicated with physicians and caregivers but are not easily recognized by researchers because (a) these concerns have not been passed along to the research community, and (b) from a research perspective, parents' observations need to be confirmed by scientific methods. One way to address this critical gap is to engage patient advocates, physicians, and outcomes researchers when designing genetics research studies, using protocols such as the one developed in the AutGO initiative, as schematically illustrated in Figure 4A/B.

The need to “bridge the translation gap” between the basic and clinical sciences in ASD has been discussed in an editorial by Szatmari, Charman, and Constantino [2012]. One essential barrier identified by the authors was the fact that translational aspects are usually added toward the end of studies instead of being identified and incorporated at the study conception phase. This barrier is particularly relevant to most, if not all, genetics research studies aimed at the discovery of risk genes. Another key roadblock in fortifying translational aspects of autism research is insufficient community engagement in the research process [Pellicano, Dinsmore, & Charman, 2014a; Pellicano, Dinsmore, & Charman, 2014b; Fletcher‐Watson et al., 2019]. A large‐scale survey conducted in the United Kingdom investigated community involvement from both researchers and community members' perspectives [Pellicano et al., 2014a; Pellicano et al., 2014b]. This semi‐structured qualitative focus group study highlights the lack of active involvement of community members in the autism research process, as well as disconnection between the current autism funding landscape and the type of questions that people with autism and caregivers would like to see addressed. In order to promote the type of research that makes a meaningful difference in the lives of those affected by autism, it is vital to have a constructive dialogue about research priorities between researchers and the autism community.

Addressing these concerns requires applying a different approach than what is being traditionally used by the genetics research community. AutGO was developed with the intention to contribute to filling this gap, and the rationale for it is summarized in Figure S3A. Some research studies use mixed methods, but typically, genetics research studies rely on quantitative methods, which include testing a hypothesis, mainly using numeric data points, and applying statistical tests to compare data, with the main objective of understanding disease etiology. On the other hand, in outcomes research, the most commonly used methods are qualitative. These methods include processing participants' perspectives using techniques such as surveys, focus groups, and interviews, and evaluating the identified themes to help build a hypothesis. Typically, the main objective of outcomes research studies is to empower patients by engaging them throughout the study. In AutGO, we aimed to develop a hybrid concept that would utilize both qualitative and quantitative approaches and their underlying principles to facilitate developing genetics research projects that are more patient‐centered. As a result, it may help better address the existing challenges in autism genetics research, including the clinical heterogeneity that negatively impacts the reproducibility of research findings.

As shown in Figure S3B, despite differences between the two research approaches in terms of hypothesis generation, collecting evidence, and/or overall objectives, their common purpose is to make research more translational, either through improving health outcomes or contributing to precision medicine. Currently, autism genetics research mainly utilizes quantitative methods, and in the AutGO initiative, we attempted to show that adding qualitative methods, that is, including patient perspectives and clinical observations in study design, may facilitate moving the field closer to translational research by fostering the formation of GO projects.

Building a partnership with a wide range of stakeholders, including genetics researchers and patient advocates, is needed in order to develop a GO project. Traditionally, outcomes research focuses on identifying and prioritizing patient needs, however, addressing those needs may be beyond the scope of genetics research. This incompatibility has been one of the key obstacles that prevented the formation of such partnerships. One solution is to build partnerships based on flexible and mixed methods, such as actionable GO projects. The approach presented in AutGO addresses this challenge by retrospectively synthesizing research findings from the literature, identifying potential future GO projects that might arise from these findings (i.e., more likely to be actionable), and prioritizing them with input from patients and other stakeholders.

AutGO is an evolving initiative, and here we describe the development of its three essential components: conceptual framework, applicability, and implementation. As a proof‐of‐concept, the AutGO team demonstrated how a GO hypothesis could be developed using a semi‐structured literature review workflow that engaged all stakeholders. To illustrate the GO concept, we also developed a prototype (i.e., *MET* discovery) from published reports and formulated one GO hypothesis for autism. We hope that the presented workflow, prototype, and the recommendations discussed below will bring awareness in the autism research community about the benefits of applying the GO approach in order to promote translational aspects in genetics research.

### *Lessons Learned*

#### *Recognizing the importance of developing a conceptual framework*

Conceptual framework development (conceptualization) is one of the essential components of building new multidisciplinary initiatives. Its importance and value, particularly, for integrative research approaches, are gaining more attention. Recently, a conceptual framework for conducting Genomic Medicine Integrative Research (GMIR) has been developed by a multidisciplinary collaborative team through literature review, consensus‐building discussions, and multiple rounds of prioritization, to aid the genomics community in developing research questions, strategies, and measures to facilitate the implementation of genomic testing in diverse clinical settings and populations [Horowitz et al., 2019]. The GMIR framework highlights the need to build a unified conceptual framework that organizes all critical research aspects such as discovery, translational, and implementation, as a major necessary step toward improving the overall mission of genomic medicine and prevention. While the GMIR provides an overall platform and justification for connecting different efforts involved in genetic research, the AutGO initiative provides a framework for one particular step, that is, how to engage stakeholders in hypothesis development. We believe that expanding engagement initiatives such as AutGO will eventually contribute toward reaching the overall goals outlined by GMIR and similar efforts.

Despite the growing attention, the importance of conceptualization is still not fully appreciated in some areas of research. Particularly, conceptualization is not a commonly targeted study objective in genetics. Throughout the study, we reminded our members that the study goal was to develop a conceptual framework by including it in every team communication. We learned that this continuous reminder was appreciated by the AutGO members and helped them remain focused on the study goal and provide perspectives accordingly.

#### *Standardizing GO literature review process*

Research findings could be interpreted and synthesized differently based on readers' background and perspectives. When working with a multidisciplinary team, having diverse points of view may be either productive or counterproductive, which in case of developing a GO hypothesis could influence the outcome of the literature review process. During the AutGO literature review process, we learned that such obstacles may be reduced if firm requirements regarding the process are defined and followed from the beginning, for example, specific inclusion criteria and evaluation metrics.

#### *Ensuring practicality and time efficiency of multidisciplinary participatory projects*

Potential challenges to this as well as other multidisciplinary activities include the real or perceived imbalance of power between study participants and participants' time constraints, as previously described [Talebizadeh et al., 2018]. To maintain a meaningful and sustainable engagement of members in a multidisciplinary participatory project, it is important to minimize the unnecessary workload on participants. To do so, we created and used uniform templates for communications and feedback collection, which made the literature review process more time‐efficient for study participants. For example, utilizing a uniformly structured format for GO topic summaries (see Supplemental File for an example) effectively served this purpose in AutGO.

#### *Recognizing the importance of educational GO examples*

Previously, we noted that reviewing practical research examples would facilitate a better understanding of novel integrated topics such as GO [Talebizadeh, Shah et al., 2018]. Here, we presented from GO perspective an actual research example, that is, the *MET* discovery, as a prototype, to illustrate how this type of integrated projects may be developed. AutGO team members found the prototype to be helpful in demonstrating the hybrid nature of the GO concept.

### *Suggested Recommendations for the Research Community*

#### *Assessing literature from the GO concept perspective*

The integrated GO concept is novel, but its important meaning may already be reflected, at least partially, in some research discoveries such as the findings discussed in the prototype. Assessing literature from the GO concept perspective is one impactful way to promote developing GO projects. Such assessments will allow identifying studies that may include GO aspects and detecting existing barriers, gaps, and opportunities for developing new GO projects.

While genetic risk factors have not been frequently assessed in relation to improving patient outcomes in autism, this type of evaluation is widely applied in other complex conditions. A quick PubMed search using the search term “single nucleotide polymorphism (SNP)” and “patient outcomes” returns several thousands of papers, indicating that a large amount of genetic data evaluating the effect of SNPs on clinical outcomes has already been generated by researchers. The majority of the published data are related to cancers or cardiovascular diseases (e.g., using SNPs for predicting the prognosis [Choi et al., 2013, Jeon et al., 2013, Rinella et al., 2013, Qi et al., 2014], treatment outcomes [Keane et al., 2013; Offer et al., 2013; Scartozzi et al., 2013], and risk assessment [Pazik et al., 2013; Zheng et al., 2013; Romanos et al., 2014; Xie et al., 2014]). While the ultimate impact of some of these findings on health outcomes remains to be elucidated, identifying genetic risk factors may have the potential to improve subtyping, and help find high‐risk patient subgroups requiring a closer follow up [Hsu et al., 2013]. It would be intriguing to see how experiences gained from incorporating patient perspectives and genetic risk factors to direct decision making in clinical practice in other conditions (oncology and cardiology) could be applied to develop successful patient outcomes predictors for autism.

#### *Developing and employing synergistic strategies for patient‐centered initiatives*

During the course of the AutGO project, through evaluating feedback from study participants and interacting with members of local and national autism communities, we became aware of a high level of interest in this endeavor and a need to find a sustainable platform for developing and promoting GO projects. While there is a consensus among different stakeholders on the need to build a bridge between outcomes and genetics research, there is a paucity of a practical and meaningful platform to implement this much‐needed synergy. The overall AutGO goal fits well with some of the ongoing patient‐centered initiatives, for example, relevant PCORI funded projects, patient advocacy efforts, and legislative and pharmaceutical initiatives (see Supplemental Fil**e** for more details). Progress in making autism genetics research more patient‐centered could be accelerated if a meaningful connection is made between these dispersed efforts. In order to direct available resources/efforts toward this goal, multiple players and policymakers need to be actively engaged in the dialogue and the process of developing a sustainable platform for promoting GO projects, which might be achieved through synergistic engagement and dissemination activities including organizing targeted educational conferences and developing educational curriculums, such as continuing medical education courses.

## Limitations

One of the main challenges in developing a conceptual framework for building a GO research hypothesis was the absence of a preexisting template. Our team has attempted to build such a framework for the first time. To do so, we used an iterative process that included drafting a stepwise protocol, finalizing it by incorporating feedback from team members, applying each step, and sharing the results with the team. As with any new initiative, we faced certain limitations while developing the AutGO conceptual framework, as described below.

With the exception of two in‐person meetings with local members, communication with the entire team was mainly done by e‐mail, including distributing topic summaries, surveys, and regular updates. While this might be viewed as a limitation, this communication method was chosen for the following reasons. As we previously noted, a potential challenge to this type of multidisciplinary engagement effort is a real or perceived imbalance of power between study participants, particularly, technical and nontechnical members. Communication by e‐mail allowed us to reduce this hurdle by providing an equal opportunity for all members to participate and contribute, regardless of their background. It also ensured that members formulated their perspectives independently, without being influenced by other participants' opinion, and their input was collected in an unbiased manner.

Busy schedules of members imposed another inevitable challenge for active engagement, meaningful contribution, and full participant retention throughout the study. In this regard, the applied communication method provided a convenient and time‐efficient way for all members to stay informed and engaged. Using a uniform format for all email updates and GO summaries further facilitated communication and members' contribution.

In summary, the present study was a proof‐of‐concept endeavor with a primary goal of developing a conceptual framework, which could be modified and/or expanded. For example, we used a two‐Tier assessment for feasibility, but more evaluation steps could be included in future studies. Also, we developed a GO hypothesis for “depression in autism,” a topic prioritized by the AutGO team; other GO teams may come up with different topics, depending on their team composition and priorities.

## Funding

This study was funded by the Patient‐Centered Outcomes Research Institute (PCORI; Contract numbers: EAIN‐2419 and EAIN‐3885). All statements in this report, including its findings and conclusions, are solely those of the authors and do not necessarily represent the views of the PCORI, its Board of Governors, or the Methodology Committee.

## Conflict of Interest

ZT, AS, and AutGO Working Group members (DS, CB, DB, SB, JB, ABE, AB, BC, MDG, MAH, VH, MI, MK, AK, KL, MM, JM, JJM, MM, CN, GN, BS, KS, MS, CS, HS, OJV, and DW) declare that they have no conflict of interest and/or nonfinancial conflicts.

## Author Contributions

Zohreh Talebizadeh and Ayten Shah prepared the first draft of the manuscript. All authors (AutGO Working Group) equally contributed to the conduct of the study, reviewed, and revised the manuscript and approved the final version.

## Supporting information


**Appendix** **S1**: Supporting Information

## Appendix 1 “AutGO Working Group” Participants (in alphabetical order):

Dustin Baldridge, MD, PhD^1^

Courtney Berrios, MSc, ScM, CGC^2^

David Beversdorf, MD^3^

Seth Bittker, BS^4^

Jeff Blackwood, MBA^5^

Andrea Bradley‐Ewing, MPA, MA^2^

Amy Brower, PhD^6^

Broderick Crawford, BS^4^

Meredith Dreyer Gillette, PhD^2,7^

Mary Anne Hammond, BS^2^

Valerie Hu, PhD^8^

Monirul Islam, MD^9^

Mary Kinart, BS^2^

Angela (Angie) Knackstedt, BSN, RN‐BC^2^

Kiely Law, MD^10^

Matthew Mclaughlin, MD, MSB^2,7^

James McClay, MD, MS^11^

Jacob J. Michaelson, PhD^12^

Matthew Mosconi, PhD^13^

Cy Nadler, PhD^2,7^

Ginger Nicol, MD^1^

Brenda Salley, PhD^14^

Kim Smolderen, PhD^7^

Matt Spence, PhD^3^

Christina Stephan, MD, PhD^4^

Holly Stessman, PhD^15^

Olivia J. Veatch, PhD^16^

Darcy Weidemann, MD, MHS^2,7^

^1^Washington University in St. Louis, St. Louis, MO

^2^Children’s Mercy Hospital, Kansas City, MO

^3^University of Missouri, Columbia, MO

^4^Patient/Parent representative

^5^Pathfinder Health Innovations, Kansas City, MO

^6^American College of Medical Genetics, Bethesda, MD

^7^University of Missouri‐Kansas City School of Medicine, Kansas City, MO

^8^George Washington University, Washington, DC

^9^Medical College of Georgia, Augusta, GA

^10^Johns Hopkins Bloomberg School of Public Health, Baltimore, MD

^11^University of Nebraska Medical Center, Omaha, NE

^12^The University of Iowa, Iowa City, IA

^13^University of Kansas, Lawrence, KS

^14^University of Kansas Medical Center, Kansas City, KS

^15^Creighton University, Omaha, NE

^16^University of Pennsylvania, Philadelphia, PA
