# Supplementary material for: The AutGO Initiative: A Conceptual Framework for Developing Genetics‐Outcomes Research Hypotheses
Source: Autism Res. 2020 Jul 3;13(8):1286–99. doi: 10.1002/aur.2331 (PMC7496490; doi:10.1002/aur.2331)

The AutGO Initiative: A Conceptual Framework for Developing Genetics-Outcomes Research Hypotheses

Zohreh Talebizadeh[1,2], Ayten Shah[1] and "AutGO Working Group"

Supplemental files include:

- **Table S1**. AB participants' background/expertise
- **Table S2.** COREQ checklist
- **Table S3.** Workshop attendees' demographic
- **Table S4.** Workshop survey results

- **Figure S1.** AB and EP
- **Figure S2.** GO hypothesis development workflow
- **Figure S3.** Comparison between qualitative and quantitative methods

- Workshop attendees' direct quotes
- AB members' direct quotes (satisfaction)
- Other initiatives
- Tier-I summary (example)
- Tier-II summary (example)
- Developed GO hypothesis

Table S1. Study Participants (AutGO Team)

Name	Initials	Expert Panel (EP)	Stakeholder Category: Parents*	Clinicians	Res-Genetics	Res-Outcomes	Res-Autism	Others	Phase I[a]	KC workshop[b]	Additional expertise
Zohreh Talebizadeh, PhD	ZT	EP-I			X	X	X		X	X	Epigentics, ASD subtyping
Ayten Shah, BSN	AS				X	X	X		X	X	
**AutGO Working Group**											
Dustin Baldridge, MD, PhD	DB1			X	X		X			X	Bioinformatics
Courtney Berrios, MSc, ScM, CGC	CB				X						Genetic counseling
David Beversdorf, MD	DB2	EP-II		X			X			X	Gene-environment interactions
Seth Bittker, BS	SB	EP-I	X						X	X	Patient advocate
Jeff Blackwood, MBA	JB							X	X	X	Industry representative-Pathfinder HI
Andrea Bradley-Ewing, MPA, MA	ABE					X		X	X	X	Community engagement leader
Amy Brower, PhD	AB		X		X			X	X	X	ACMG-NBSTRN, project manager
Broderick Crawford, BS	BC							X	X	X	Faith community engagement advocate/leader
Meredith Dreyer Gillette, PhD	MDG			X			X				Psychologist-obesity & weight management
Mary Anne Hammond, BS	MAH		X					X	X	X	Patient & community advocate, Education coordinator
Valerie Hu, PhD	VH	EP-I	X		X		X		X	X	Epigentics, ASD subtyping
Monirul Islam, MD, PhD	MI			X		X			X	X	Epidemiologist
Mary Kinart, BS	MK		X						X	X	Physician recruiter
Angela Knackstedt, BSN, RN-BC	AK							X	X	X	Health literacy coordinator
Kiely Law, MD	KL		X	X		X	X			X	Interactive Autism Network (IAN)-Research director
Matthew McLaughlin, MD	MM			X					X	X	Physical medicine & rehabilitation
Jim McClay, MD, PhD	JM			X		X		X	X	X	PCORnet, electronic medical records, biorepository
Jacob J Michaelson, PhD	JJM	EP-II			X		X	X		X	Computational biology, Predictive models
Mattew W Mosconi, PhD	MWM			X			X			X	Psychologist-sensorimotor problems
Cy Nadler, PhD	CN	EP-II		X			X			X	Psychologist-ASD early detection & intervention
Ginger Nicol, MD	GN			X			X		X	X	Psychiatry-obesity & weight management
Brenda Salley, PhD	BS			X			X				Psychologist-ASD early detection & intervention
Kim Smolderen, PhD	KS					X			X		Psychology-cardiovascular disorders
Matt Spence, BS	MS				X			X		X	Bioinformatics
Christina Stephan, MD, PhD	CS			X				X			Industry representative-Health Story Bank, Inc
Holly Stessman, PhD	HS	EP-II			X		X				Functional genomic, ASD subtyping
Olivia Veatch, PhD	OV	EP-I			X		X		X	X	Sleep disorders, ASD subtyping
Darcy Weidemann, MD, MHS	DW			X					X		Nephrology
		**Total (N)[c]**	**7**	**13**	**10**	**7**	**14**	**10**	**18**	**23**	
		**Total (%)**	**23%**	**43%**	**33%**	**23%**	**47%**	**33%**	**60%**	**77%**	

***Parents:** We had a total of 7 parent representatives. One of them requested to remain anonymous and is not shown in this table.

[a]**Phase I:** Participated in AutGO Phase I
[b]**KC Workshop:** Attended the AutGO-KC workshop
[c]The total N was calculated based on the total number of participants (N=30). Of note, some CAB members selected more than one disease category.

Table S2. Consolidated criteria for reporting qualitative studies (COREQ): 32-item checklist (recommended by Tong et al., 2007)-AutGO project-Phase II

No Item	Giude questions/description	AutGO project-Phase II
**Domain 1: Research team and reflexivity**		
**Personal Characteristics**		
1. Interviewer/facilitator	Which author/s conducted the interview or focus group?	ZT (Principle Investigator-PI) & AS
2. Credentials	What were the researcher's credentials? *E.g. PhD, MD*	PhD & BS
3. Occupation	What was their occupation at the time of the study?	Scientist (Geneticist) & Research Assistant
4. Gender	Was the researcher male or female?	Females
5. Experience and training	What experience or training did the researcher have?	ZT (17 years of experience in conducting genetics research as a PI plus 5 years of experience with developing outcomes research projects), AS (5 years of experience contributing to both genetics and outcomes research projects)
**Relationship with participants**		
6. Relationship established	Was a relationship established prior to study commencement?	The establishment of relationship has been an evolving process and began prior to study commencement. ZT, SB, AS, and KS developed the original research plan. OV and VH were involved in further refinement of the research plan and designing a community outreach protocol. Eighteen members were involved from inception of AutGO and took part in Phase I. Through their networks, twelve new members were recruited for Phase II. See Table S1 for more details on participants' background/expertise.
7. Participant knowledge of the interviewer	What did the participants know about the researcher? *e.g. personal goals, reasons for doing the research*	All study participants were informed about the PI's areas of research and the goals and expectations for doing this outcomes research. They were also informed about other participants' background in relation to the present study. For example, a summary of each participants' expertise, experiences, and role in the study (brief biography) was distributed among all members during in-person meetings and through email communications.
8. Interviewer characteristics	What characteristics were reported about the interviewer/facilitator? *e.g. Bias, assumptions, reasons and interests in the research topic*	Both interviewers/facilitators were interested on the overall goal of the AutGO study. However, their approaches/input were rather complementary. ZT's focus was tailored toward ensuring adequate engagement of the AutGO members throughout the study process, dissemination plans, and finding applicable ways to communicate the identified results to the autism research community. AS focused on building surveys and processing participants' feedback.
**Domain 2: study design**		
**Theoretical framework**		
9. Methodological orientation and theory	What methodological orientation was stated to underpin the study? *e.g. grounded theory, discourse analysis, ethnography, phenomenology, content analysis*	Dialogue type approach (e.g., in-person meetings, emails, phone calls) was used by the PI to directly communicate study updates, participants' feedback, and tasks with the study participants. Semi-structured methods (i.e., survey links distributed via emails as well as in-person meetings) were used to obtain perspectives and views of study participants.
**Participant selection**		
10. Sampling	How were participants selected? *e.g. purposive, convenience, consecutive, snowball*	See the above description for the item No.6. To reach study goals, participants were selected/invited purposively to form a multidisciplinary team, including representatives from the following stakeholder groups: outcomes researchers, genetics researchers, clinicians, parents/patient representatives, as well as community and industry representatives.
11. Method of approach	How were participants approached? *e.g. face-to-face, telephone, mail, email*	Face-to-face, email, telephone
12. Sample size	How many participants were in the study?	30 members
13. Non-participation	How many people refused to participate or dropped out? Reasons?	None
**Setting**		
14. Setting of data collection	Where was the data collected? *e.g. home, clinic, workplace*	Local members met at the Children's Mercy main campus, when applicable. Online surveys were taken at members convenience.
15. Presence of nonparticipants	Was anyone else present besides the participants and researchers?	Some workshop attendees (nonparticipants) provided feedback, and their comments were considered in developing the study protocol and crafting future implentation strategies.
16. Description of sample	What are the important characteristics of the sample? *e.g. demographic data, date*	Study participants' background and stakeholder categories that they represented are included in Table S1. Demographic data (gender, age, race, and education level) for Workshop attendees are provided in Table 1.
**Data collection**		
17. Interview guide	Were questions, prompts, guides provided by the authors? Was it pilot tested?	Two sets of survey (Tier-I and Tier-II surveys) were created by ZT and AS. When applicable, ABE and OV were consulted on finalizing the surveys. All surveys were distributed through SurveyMonkey among all participants.
18. Repeat interviews	Were repeat interviews carried out? If yes, how many?	Ten potential GO topics were scored by study participants in Tier-I based on their perspectives (i.e., individual experience and opinion) . Top three topics were prioritized in Tier-II based on the same criteria.
19. Audio/visual recording	Did the research use audio or visual recording to collect the data?	Yes, in-person meetings were audiorecorded.
20. Field notes	Were field notes made during and/or after the inter view or focus group?	Audio recordings of in-person meetings were transcribed by AS and revewied/finalized by ZT. Notes were also taken by ZT and AS during each meeting.
21. Duration	What was the duration of the interviews or focus group?	About 2 hours for each in-person meeting and varied for other communication means (email and phone conversations).
22. Data saturation	Was data saturation discussed?	N/A
23. Transcripts returned	Were transcripts returned to participants for comment and/or correction?	Minutes of meetings and feedback obtained via surveys were shared with the study participants by email communications.

Continued---next page

Domain 3: analysis and findings		
**Data analysis**		
24. Number of data coders	How many data coders coded the data?	Two coders (ZT and AS) coded the data.
25. Description of the coding tree	Did authors provide a description of the coding tree?	For the literature review process the following inclusion criteria were applied: Papers must include at least one of the following 3 elements: patient health outcome(s), genetic risk factor(s), or combination of both.<br>Papers must be related to one of the following conditions: autism, other neurodevelopmental disorders, or co-morbid conditions (i.e., symptoms that co-occur with autism such as sleep problems, eating problems, etc.).
26. Derivation of themes	Were themes identified in advance or derived from the data?	Nominating papers for team evaluation was an optional activity. Seven AutGO members (SB, AB, VH, AK, JJM, OV, and AS) recommended 60 papers for team evaluation. ZT and AS independently reviewed the recommended papers, with each paper being reviewed twice for accuracy. Together, ZT and AS synthesized the information from the literature to narrow the papers down by themes and summarized ten potential GO topics. All study members (n=30) participated in Tier-I and Tier-2 assessments by reviewing and ranking the GO topic summaries. See Figure S2 for the identified themes (GO topics). Based on the inclusion criteria listed in the item No.25, some of the main papers recommended by the members, were related to patient health outcome(s) and did not include genetics. In Tier I summary, in addition to a brief description of a main paper, potential direction for developing a GO hypothesis around the selected topic (e.g., probiotics or CBT) was outlined by including information from additional papers along with the PubMed IDs. This approach was further extended in Tire II to demonstrate how a potential GO hypothesis could be developed. An example of GO topic summaries for the top prioritized topic (depression) as well as the developed GO hypothesis are provided in the Supplement File, which exemplify how information from literature was synthesized and communicated with the team members to obtain their feedback in Tier I and Tier II evaluations.
27. Software	What software, if applicable, was used to manage the data?	Not applicable
28. Participant checking	Did participants provide feedback on the findings?	The GO topic summaries were shared with study participants and their feedback was collected through project surveys.
**Reporting**		
29. Quotations presented	Were participant quotations presented to illustrate the themes/findings? Was each quotation identified? *e.g. participant number*	Yes, examples of participant quotations were provided in the Supplemental file.
30. Data and findings consistent	Was there consistency between the data presented and the findings?	This was a conceptual development project and the information shared with participants, especially, in GO topic summaries were accompanied by supporting literature (i.e., PubMed ID).
31. Clarity of major themes	Were major themes clearly presented in the findings?	To minimize unnecessary workload on participants and for clarity, we utilized uniformly structured format for GO topic summaries (see Supplemental File for an example).
32. Clarity of minor themes	Is there a description of diverse cases or discussion of minor themes?	In Tier-II, ZT and AS conducted an additional literature search for each topic using the following search terms, while replacing 'topic' with the respective terms provided in Figure S2: "gene & topic", "outcomes & topic", "autism & topic", "treatment & topic", "dietary supplementation & topic", and "neurodevelopmental disorders & topic". The information obtained from this targeted literature search was then used to identify a few relevant areas, or sub-topics (i.e., minor themes), that might be considered in developing a GO hypothesis. A brief justification of why/how a sub-topic could be related to building a GO hypothesis was also included (i.e., take home messages). As shown in Tables 1 and 2, some variations were found in prioritizing GO topics among stakeholder groups. Identifying if/why given topics might be perceived differently across stakeholder groups was beyond the scope of this study. Assessing such topics would require a larger sample size and different study design.

Table S3. Demographic data-AutGO-KC Workshop attendees

Gender	N[a]	%
Female	26	54
Male	22	46
**Race**		
White/Caucasian	34	70.8
Black/African American	5	10.4
Asian	7	14.6
Other race (Mixed race and Hispanic)	2	4.2
**Education level**		
Bachelor's degree	9	18.8
Master's degree	7	14.6
Professional/Doctorate degree	32	66.7
**Stakeholder Category***		
Parents/Family members of individuals with ASD	10	20.8
Clinicians (i.e., medical, psychology)	14	29.2
PCORI funded Investigators	6	12.5
Outcomes researchers	6	12.5
Genetics researchers	11	22.9
Autism researchers	15	31.3
Scientists (other fields)	21	43.8
Others**	18	37.5

[a] Based on responses of 48 participants who took the survey, out of total 60 attendees (all 60 participants attended the workshop in-person)

*Some attendees represented more than one stakeholder category

**Others: 4 Research Administrators, 4 Community Leaders, 4 Bioinformaticians, 2 Bioethics experts, 2 Industry Representatives, 2 Funding Agency representatives

Table S4. Average rating of AutGO-KC Workshop attendees' personal perspectives on topics discussed (n=48 survey respondents)

#	Topics/Questions	Stakeholder Category								*M (SD)
		Parents/Family members of individual with ASD	Clinicians (i.e., medical, psychology)	PCORI funded Investigators	Outcomes researchers	Genetics researchers	Autism researchers	Scientists (other fields)	Others	
0	"I find the main topic of the workshop (i.e., Incorporating genetics into PCOR) of interest."	9.6 (0.7)	9.1 (1.2)	8.8 (1.8)	8.7 (1.5)	9.5 (0.8)	9.3 (1.0)	9.3 (1.1)	9.3 (1.0)	9.2 (1.0)
2	"I think there is a need to shift/change the culture in autism genetic research from being researcher-driven to becoming more patient-centered."	9.0 (1.2)	8.1 (1.7)	9.3 (0.8)	8.8 (1.6)	7.8 (1.9)	8.4 (1.7)	8.5 (1.7)	8.7 (1.2)	8.6 (0.8)
3	"I think there is a need to incorporate patients/parents/caregivers' perspectives into research design, to make autism genetic research more patient-centered."	9.5 (0.8)	8.6 (1.5)	9.0 (1.3)	9.3 (0.8)	8.6 (1.4)	8.9 (1.3)	8.9 (1.3)	9.3 (1.0)	9.0 (0.6)
4	"I think there is a need to establish collaborations between outcomes and genetics researchers, to make autism genetic research more patient-centered."	9.4 (0.8)	8.7 (1.0)	8.0 (2.1)	9.0 (2.0)	8.5 (1.2)	8.9 (1.1)	9.0 (1.0)	8.9 (1.3)	8.8 (0.9)
5	"I think this kind of educational workshops may contribute to building a bridge between outcomes and genetics research communities."	8.8 (1.7)	8.2 (1.3)	7.2 (3.7)	7.7 (3.9)	8.5 (1.6)	8.5 (1.5)	8.8 (1.4)	8.7 (1.7)	8.3 (1.4)
6	"I think the PCORI AutGO workshop-1 provided an opportunity for attendees to learn about patient-centered research"	9.6 (0.7)	9.0 (1.1)	6.8 (2.9)	7.2 (3.3)	8.9 (1.3)	8.6 (1.4)	8.7 (1.5)	8. 8 (1.3)	8.4 (1.2)
7	"I think the PCORI AutGO workshop-1 provided an opportunity for attendees to build collaborations"	8.3 (2.1)	8.2 (1.5)	6.7 (3.3)	7.0 (3.6)	8.2 (2.2)	7.7 (2.0)	8.2 (1.9)	7.9 (2.3)	7.8 (1.5)
8	"I think the PCORI AutGO workshop-1 provided an opportunity for attendees to stimulate new research ideas"	8.7 (1.6)	8.4 (1.5)	5.8 (3.1)	7.0 (3.8)	8.5 (2.2)	7.9 (2.0)	8.5 (1.7)	8.2 (1.6)	7.9 (1.4)
9	As a result of attending this conference, have you learned something new about PCORI mission?	9.3 (0.9)	8.6 (1.6)	5.3 (2.9)	5.8 (3.8)	8.4 (1.5)	8.4 (1.8)	8.3 (2.0)	7.5 (2.1)	7.7 (1.5)
10	As a result of attending this conference, have you learned something new about PCOR?	9.3 (0.9)	8.3 (1.6)	5.5 (3.0)	5.7 (3.6)	8.5 (1.4)	8.2 (2.0)	8.3 (2.0)	7.8 (2.4)	7.7 (1.5)
11	As a result of attending this conference, have you learned something new about incorporating genetic information into PCOR?	9.3 (0.8)	8.5 (1.3)	7.2 (2.3)	8.2 (2.7)	8.5 (1.6)	8.5 (1.4)	8.9 (1.2)	8.5 (1.4)	8.4 (1.1)
12	As a result of attending this conference, have you learned something new about developing patient-centered genetic research questions/hypotheses/	9.1 (0.9)	8.4 (1.1)	7.3 (2.3)	8.0 (2.6)	8.5 (1.6)	8.3 (1.5)	8.6 (1.4)	7.8 (1.4)	8.2 (1.1)
13	How informative the presentation on PCORI mission and patient engagement in research was?	9.0 (1.6)	8.6 (0.8)	7.7 (1.6)	7.8 (1.6)	8.8 (1.2)	8.6 (0.9)	8.5 (1.2)	8.4 (1.1)	8.4 (0.7)
14	How informative the presentation on the AutGO project, goals and methods was?	8.8 (1.6)	8.2 (0.9)	8.5 (1.2)	8.3 (1.0)	8.6 (1.4)	8.7 (1.1)	8.6 (1.2)	8.5 (1.1)	8.5 (0.6)
15	How informative the presentation on existing genetic/phenotypic resources was?	9.3 (0.9)	8.1 (1.2)	8.3 (1.6)	8.7 (1.4)	8.9 (1.1)	8.6 (1.2)	8.8 (1.3)	8.4 (1.3)	8.6 (0.7)
16	How informative the presentations on parents/caregivers/patients perspectives were?	9.4 (0.7)	8.4 (1.0)	7.2 (3.1)	7.0 (3.1)	9.3 (0.9)	9.1 (1.1)	8.8 (1.4)	9.1 (1.1)	8.5 (1.1.)
17	How beneficial the perspectives of parents/ caregivers/patients were to you?	9.6 (0.5)	8.7 (1.1)	9.0 (1.1)	9.0 (1.3)	9.2 (1.0)	9.2 (0.9)	8.9 (1.3)	9.3 (0.8)	9.1 (0.6)
18	How beneficial the perspectives of clinicians were to you?	9.2 (0.6)	7.9 (1.5)	7.7 (1.6)	7.3 (1.4)	8.8 (1.1)	8.5 (1.1)	8.4 (1.3)	8.3 (0.9)	8.3 (0.7)
19	How beneficial the perspectives of genetics researchers were to you?	9.2 (0.7)	8.3 (1.3)	7.2 (2.0)	7.3 (2.0)	8.9 (1.0)	8.7 (0.9)	8.6 (1.2)	8.2 (1.1)	8.3 (0.8)
20	How beneficial the perspectives of outcomes researchers were to you?	9.0 (0.8)	8.2 (1.2)	8.0 (1.5)	7.8 (1.5)	8.9 (1.0)	8.6 (1.2)	8.5 (1.3)	8.2 (1.2)	8.4 (0.7)
21	How beneficial the perspectives of research community representatives (panel 3) were to you?	9.2 (0.7)	8.1 (1.2)	7.8 (1.3)	7.7 (1.2)	9.1 (1.0)	8.7 (1.1)	8.7 (1.1)	8.3 (0.8)	8.4 (0.6)
22	How beneficial the perspectives of KC community representatives (panel 4) were to you?	9.1 (0.8)	8.2 (0.7)	7.5 (1.5)	7.5 (1.5)	8.8 (1.1)	8.1 (1.8)	8.2 (1.7)	7.8 (1.7)	8.2 (1.0)
23	How satisfied you were with the overall flow of the workshop?	8.7 (1.3)	7.9 (1.5)	6.3 (3.1)	6.3 (3.2)	8.4 (1.6)	8.0 (1.4)	8.2 (1.6)	7.4 (2.0)	7.7 (1.3)
24	How satisfied you were with selection of speakers?	9.2 (1.2)	8.5 (1.3)	7.8 (1.7)	8.0 (1.9)	9.1 (0.9)	8.9 (1.2)	8.8 (1.2)	8.4 (1.1)	8.6 (0.7)
25	How satisfied you were with video messages?	8.8 (1.6)	8.0 (1.6)	8.2 (2.1)	7.8 (1.9)	8.8 (1.5)	8.8 (0.9)	8.5 (1.4)	8.3 (1.2)	8.4 (0.7)
26	How satisfied you were with topics covered?	9.1 (1.3)	8.1 (1.6)	6.8 (3.3)	6.8 (3.4)	9.0 (1.0)	8.6 (1.1)	8.6 (1.5)	8.3 (1.1)	8.2 (1.1)
27	How satisfied you were with educational aspect of the workshop?	9.1 (1.0)	8.4 (1.4)	7.0 (3.3)	7.2 (3.4)	8.6 (1.6)	8.5 (1.4)	8.7 (1.2)	8.3 (1.1)	8.2 (1.2)
28	How satisfied you were with uniqueness of the topic?	9.2 (0.8)	8.7 (1.1)	9.0 (1.1)	9.2 (1.3)	9.1 (1.1)	8.8 (1.1)	8.8 (1.2)	8.3 (1.2)	8.9 (0.7)
29	How satisfied you were with multidisciplinary format?	9.3 (0.9)	8.6 (1.6)	8.2 (2.1)	8.0 (2.3)	9.0 (1.2)	8.8 (1.2)	8.8 (1.5)	8.6 (1.3)	8.7 (0.9)
30	We are planning to prepare additional educational workshops/seminars related to this topic. Would you be interested to attend/contribute to the future events?	10/10 Agree (100%)	13/14 Agree (93%)	5/6 Agree (83%)	5/6 Agree (83%)	11/11 Agree (100%)	15/15 Agree (100%)	20/21 Agree (95%)	18/18 Agree (100%)	45/48 Agree (93.8%)
31	Would you like to receive periodic email updates about our future activities (e.g., project website, other workshops/seminars, and research examples)?	10/10 Agree (100%)	12/14 Agree (86%)	5/6 Agree (83%)	5/6 Agree (83%)	10/11 Agree (91%)	14/15 Agree (93%)	19/21 Agree (90%)	17/18 Agree (94%)	42/48 Agree (87.5%)
32	Would you recommend attending this type of workshop to your colleagues/co-workers/friends?	10/10 Agree (100%)	13/14 Agree (93%)	5/6 Agree (83%)	5/6 Agree (83%)	11/11 Agree (100%)	15/15 Agree (100%)	21/21 Agree (100%)	17/18 Agree (94%)	44/48 Agree (91.7%)
33	Would you think this kind of workshop could facilitate engagement and translational research development for other disease areas?	10/10 Agree (100%)	13/14 Agree (93%)	5/6 Agree (83%)	5/6 Agree (83%)	11/11 Agree (100%)	15/15 Agree (100%)	21/21 Agree (100%)	18/18 Agree (100%)	46/48 Agree (95.8%)

Mean and SD values are provided per stakeholder groups.
*M equals the mean average across groups. SD equals the mean stdev across groups
Participants' responses are based on a scale from "0-Strongly disagree" to "10-Strongly agree"

Figure S1. Advisory board and expert panel compositions. Some participants represented more than one stakeholder category.

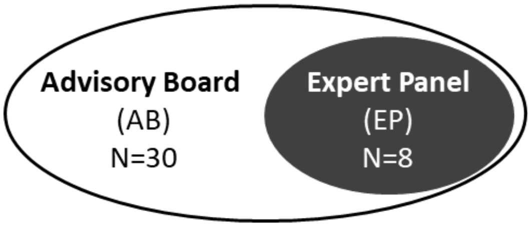

Stakeholder Category-AB	N	%
Parents/Family members of individuals with ASD	7	23%
Clinicians (i.e., medical, psychology)	8	26%
Scientists		
Autism researchers	14	47%
Genetics researchers	9	30%
Outcomes researchers	4	13%
Others	8	26%

Figure S2. GO hypothesis development workflow: steps taken and results obtained at each step are shown.

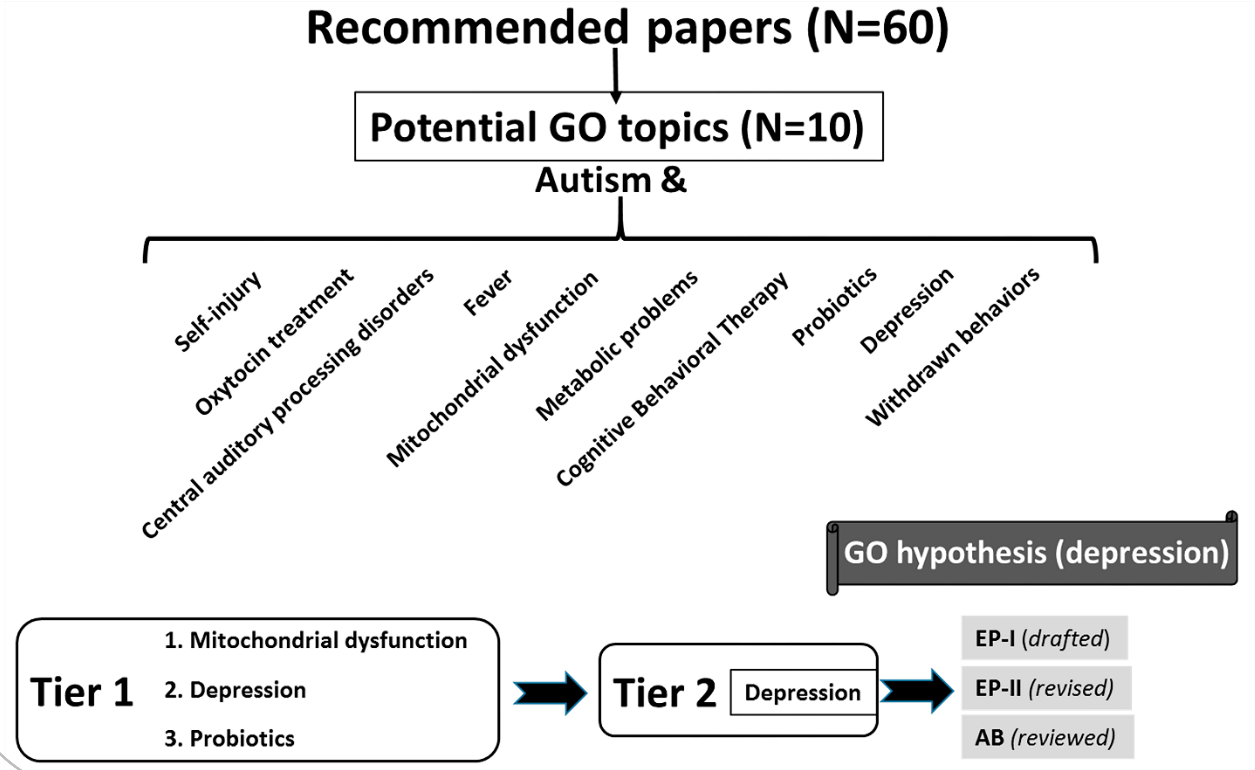

Figure S3. Comparison between qualitative and quantitative methods, in relation to the AutGO concept.

A) The existing challenges in ASD genetics research, including extensive heterogeneity and insufficient reproducibility, and the main methods used in genetics and outcomes research, are summarized. The AutGO hybrid conceptual framework is based on utilizing both methods.

B) Schematic illustrating different aspects (hypothesis, evidence, and objective) of the main analytical methods used in genetics and outcomes research (quantitative and qualitative, respectively). Despite differences in approach and directions (precision medicine and health outcomes), their ultimate common goal is to contribute to translational research. Currently, autism genetics research mainly utilizes quantitative methods (left side) and incorporating anecdotal observations using qualitative methods (right side) may help bridge the translational gap (center), as discussed in the AutGO initiative.

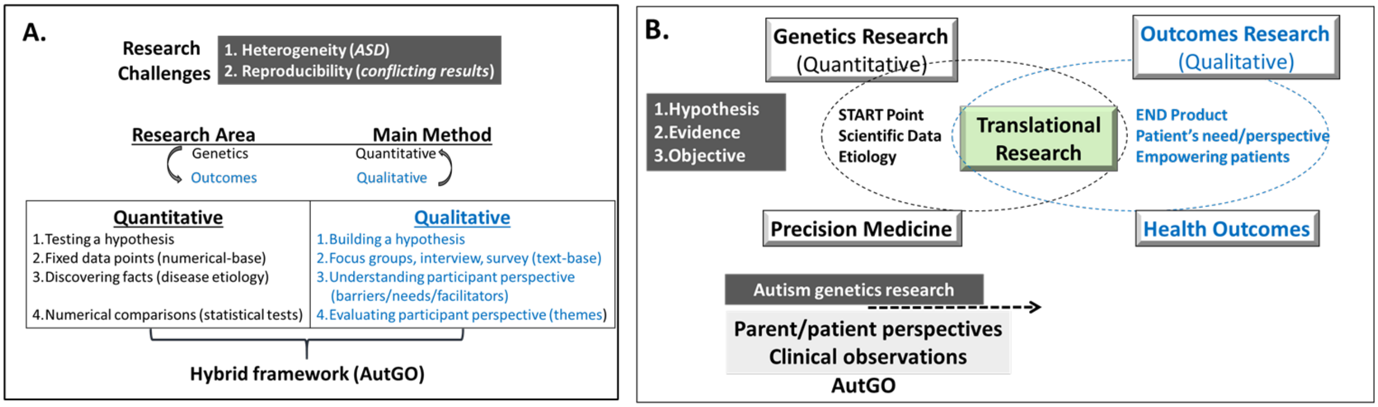

Direct Quotes from AutGO 2017 Workshop Attendees

1. Clinicians and Scientists

Psychiatrist: "It's so neat to see the momentum your passion and dedication have created. I'm very excited to be a part of your vision, and looking forward to working with you and the wonderful team you've assembled moving forward. My main takeaway was that this field is unique, this idea is extremely novel, and you have a critical mass of bright and motivated individuals that can help make it happen!"

Clinical Psychologist: "Thank YOU for this great event! The range of perspectives really did expand how I think about what "patient centered" really means, and how AutGO (love the nickname) might take shape. My main takeaway was that there is ample opportunity for interdisciplinary collaboration and stakeholder engagement!"

Scientist #1: "Communicating with patients and their families and understanding their perspectives and what they want are important in guiding the research."

Scientist #2:"Incorporating patient experience and expectations needs to get greater priority in autism research."

Scientist (clinical informatics): "More similar workshop and possible new topics related to health policy and management related to Autism clinical care, research and education at local and national level."

Scientist (computational genetics): "My main takeaway was that integration of goals of researchers with goals of families is necessary for many reasons, and should be fostered…..It was an impressive demonstration of your organizational efforts to bring such a diverse group together."

Scientist #3: "There is a strong interest in working together to solve this complex problem!"

Scientist (translational bioinformatics): "The main learning lesson for me was awareness of current genetic resources and some research areas currently explored and a sense of their success and failures."

Scientist (computational genomics): "I had a lovely time (thank you Zohreh!), and the question I keep coming back to is: when are we all going to get together again? My takeaway was that parents/caregivers are in general far more liberal in their attitudes about genetic research than the (often paternalistic) researchers are."

Scientist (autism genetics) & parent: "I think the workshop demonstrated how incorporating the patients'/families' perspectives and characteristics into genetics research could really advance genetic research towards translational goals. It was my pleasure in being a part of this innovative effort to link outcomes with genetics!"

Scientist (autism genetics):"Patient-centered research is a very important avenue for autism genetics research to follow. Engaging researchers with representatives from the autism community will be very beneficial to helping formulate more actionable research questions focused on informing precision medicine in autism."

Scientist (precision health): "Provide avenues for feedback and discussion. Make this a two-day workshop to accommodate the program."

2. Patient Advocates

Parent/Patient representative: "Thank you so much for inviting me to this conference. I was very honored that you mentioned me in your talk. In fact I almost cried. You are putting together something wonderful here. PCORI is real."

Parent/Patient representative: "There is great value in families/professionals/researchers all talking together to understand their different interests, needs and challenges and this creates a better chance of meaningful, helpful research for people impacted by the condition/disease that we are addressing. Thank you for the opportunity to represent the family challenge."
Parent/Patient representative: "It was very exciting to see talented people working to improve the lives of individuals on the Spectrum. It is much needed work. I'd extend the time for the workshop/seminar to 1 day."

Local patient advocacy group (RareKC): "Collaboration is the necessary mandate of future research and without parent/patient engagement you would be missing out on one of the strongest stakeholders/voices at the table."

Patient engagement & community leader: "Overall I thought the speakers were fantastic. I especially liked hearing parent's personal stories and perspectives of incorporating genetic data in outcomes research. The most memorable part of the workshop for me was hearing perspectives from parents. Often we think about pediatric diseases and illnesses in medical terms, without understanding the full impact on patients and families. Hearing stories directly from parents also allows the research community to identify what's missing in existing research and literature and what types of research and outcomes matter most to patients and their families."

3. Organizations and Others

Autism Simons Foundation representative: "I think you did a great job organizing the event and bringing in stakeholders that represent the full spectrum of the topic. My takeaway was the importance of including patient perspectives in genetics research."

Interactive Autism Network (IAN) representative: "It was a wonderful workshop and a great start to future work together."

Policy maker & parent: "The workshop was amazing**--**patients are important to define research objectives."

Local organization (KCALSI): "My main takeaway was that dynamic community focused on ASD is ripe for expanding collaborations."

Research institute management representative: "Thank you for organizing such a wonderful workshop! It was great to be a part of it. Multiple stakeholders in the room indicated that this sort of workshop is definitely worth doing!"

Research business operations representative: "I was one who did not know much about this area and walked away with more knowledge - and very diverse knowledge listening to the various speakers in their area of expertise. Well done!"

Direct Quotes from the AutGO members

Parent: “*I think the AutGO project was well executed. All stakeholders contributed to the development of patient-centered topics. The study team developed and presented the topics in a format that was informative and also easy to understand. The AutGO project was very successful in presenting genetic research knowledge coupled with topics of interest to patients and caregivers*.”

Clinician: “*Brought together experts from several disciplines, great opportunity to develop questions and collaborations that are directly relevant to patient outcomes*.”

Parent &Policy Maker: “*The structure of this project made participation possible for busy researchers/experts/stakeholders. I continue to feel that this is an innovative approach to collaborative research! Excellent project! So happy to be a part of it!*”

Clinician: “*As a clinical psychologist I have lots of involvement in community outreach and patient/family engagement activities – however, experiencing the AutGO process has really made me reflect on the fact that my outreach and engagement work really hasn’t been bidirectional or informed by what stakeholders are interested in to the extent that it could. … I have started to support a coalition of stakeholders (parents, self-advocates, providers, etc.) to develop a new kind of community engagement event that is a direct outgrowth of the AutGO process…..I started to understand more how truly disconnected researchers often are from the population they are trying to support. AutGO really challenged me to engage in the hypothesis-generating process in a collaborative way, and to value the perspectives of other stakeholders*."

Community leader: “*As someone who had not had much experience with genetics research, the AutGO project helped to demystify genetics research and provides a clear roadmap of how genetics and outcomes research can be combined to improve patient health outcomes*.”

Patient-Centered initiatives relevant to AutGO

Some relevant initiatives are discussed below:

PCORI supported efforts. Mental health is one of the top prioritized area of focus for PCORI, with more than 92 funded outcomes research projects for conditions such as depression, substance abuse, schizophrenia/psychotic disorders, bipolar disorder, anxiety disorders, and neurodevelopmental disorders, including autism and ADHD (https://www.pcori.org/research-results, last seen October 2019). Of 92 mental health outcomes studies, 61 (66%) are focused on treatment and the rest are related to other areas, including prevention and screening. We searched the PCORI website using a search term "autism" and found 20 funded projects, as of October 2019. These autism related projects are focused on different areas of outcomes research, such as community/stakeholders' engagement, implementation of new evidence-based treatments, improvement of currently existing intervention/treatment options, organizing educational workshops, as well as development/implementation of computerized technology to support decision making and improve outcomes for patients, families, and caregivers.

Two out of the 20 projects pertaining to the focus of our study are Interactive Autism Network (IAN) and Phelan-McDermid Syndrome Data Network (PMS_DN). IAN is a family-centered research network (www.IANresearch.org) enrolling both children and adults with ASD, along with immediate family members (more than 50,000 participants). The PMS foundation is a parent-driven organization founded by families of children diagnosed with PMS, a rare genetic condition caused by deletions of 22q13 or mutations of the SHANK3 gene and highly associated with ASD and intellectual disability. In 2013, the PMS foundation established the PMS Data Network (PMS_DN) to integrate data from the patient registry with clinical information from electronic health records of PMS patients. IAN and PMS_DN have recently leveraged their existing infrastructures to become patient-powered research networks within a PCORI infrastructure called PCORnet. Therefore, it would be interesting to see if such an integration between autism and outcomes related resources could be explored by the autism research community, as a potential avenue, for developing patient-centered projects.

AGENDA-Special Interest Group. In 2015, the Alliance for Genetic Etiologies in Neurodevelopmental Disorders and Autism (AGENDA) was formed. The mission of AGENDA was defined by seven patient advocacy and science groups that represented individuals with a known form of syndromic autism. The AGENDA research strategy is to engage notable, reliable research institutions and/or patient advocacy groups to dissemination and make science more translational. AGENDA activities include providing a collaborative platform between patient advocacy groups and researchers to promote infrastructure needs, communication with families, and coordination of efforts.

FDA (Food & Drug Administration)-Pharmaceutical Companies. In 1990s, the FDA launched its patient representative program, the purpose of which is to ensure that patient standpoints are represented in important discussions about regulatory decision-making. As of April 2018, 200 FDA patient representatives were involved in 300-500 diseases/conditions/device experiences (sources: https://www.fda.gov/media/112228/download & https://www.fda.gov/media/109891/download). Pharmaceutical companies adopted similar procedures to engage patients in the process of drug development. Currently, Roche is moving from simple engagement with patient groups towards an increased level of patient partnership in the drug development process. Similar activities are being developed in area of autism research. For example, an advisory board between Roche and individuals with autism has been formed at Autism Speaks (source: https://www.roche.com/research_and_development/who_we_are_how_we_work/clinical_trials/patient-as-partner.htm).

UK-Autism Patient Advocacy. There has been substantial expansion of public participation across the UK's National Health Service (www.invo.org.uk) towards translating basic science autism research into service provision in the past decade. For example, a group of UK researchers investigated the views of community involvement in autism research (Reference #4). The perspectives of 1,516 respondents, including autism researchers and community members such as autistic adults, family members and practitioners, were assessed in a large-scale questionnaire study to evaluate if lack of community engagement represents a barrier to the translational endeavor. The results showed the lack of a shared approach to community engagement in UK autism research. Focus groups/interviews demonstrated that while researchers were skeptical about increasing community engagement in the research process, patients expressed enthusiasm about being involved in the research process. The research group emphasized the need to develop participatory research methods in the field of autism research in order to achieve impactful outcomes. Recently, a UK seminar series have been organized to develop a framework for such collaborative efforts (Reference #5).

Background

Main paper (PMID: 29898212):

In this paper, trajectories of depressive symptoms were evaluated from childhood into early adulthood (from ages 10 to 18 years) in children with and without ASD. A series of assessments, including the short mood and feelings questionnaire, was used to establish depression diagnosis. In general, a higher prevalence of depressive symptoms was detected in children with ASD compared with controls. Furthermore, social communication impairments were associated with depression at age 18 years, and bullying explained a substantial proportion of this risk.

A potential direction for a GO project:

This paper maybe a good candidate to be considered for a GO study because of the following:

- Depression is frequently seen in subjects with autism.
- Some genetic risk factors have been identified in autism in relation to depression.
- Depressive symptoms may be measured by psychological assessments/questionnaires.

Taken together, a combination of genetic risk factors for depression, and relevant behavioral assessments may be used to identify ASD subjects who may be at a higher risk for developing depression. Specific intervention therapy may be applied for this ASD subtype to reduce depressive symptoms and social communication impairments.

Supportive papers:

PMID: 29942085
PMID: 29913130

Tier-II Summary (Example) **Topic: depression in autism**

A. Background

Depression is frequently seen in subjects with autism, but the reported prevalence varies from 1.4% to 38%. Factors contributing to this inconsistency include the lack of a gold standard assessment and measurement tool of psychiatric comorbidities* designed specifically for ASD population (**PMID:** 21502871). A longitudinal study has shown that ASD children have higher depressive symptoms scores by age 10 year, which persist to age 18 years, compared with the general population (**PMID:** 29898212). Severity of depression symptoms in children with ASD has been associated with genetic risk factors (e.g., a polymorphism in a serotonin receptor gene) (**PMID:** 24968012).

B. Sub-Topics

1. **Maternal prenatal depression** **[GENETICS & OUTCOMES]**
   a. Maternal prenatal depression has been reported as a risk factor for early-life psychopathology in children (i.e., internalizing* and externalizing* problems). Analyzing data (i.e., mother's self-reported depressive symptoms during pregnancy AND reports of psychological symptoms in their children) has shown that such association could be at least partially due to genetic factors shared between mother and child (**PMID:** 30245187).

Source: *Norwegian Mother and Child Birth Cohort study*
Sample size: 22,195 mothers and 35, 299 children

Take Home Message:
-There may be shared genetic risk factors for depression and psychopathology.

2. **Executive functions** **[GENETICS & OUTCOMES]**
   a. Depression is associated with deficits in executive functions*, a phenotypic feature frequently seen in ASD. A longitudinal twin study showed that such an association is mainly due to genetic risk factors (**PMID:** 30250762).

Sample size: 439 same-sex twin pairs

Take Home Message:
-There may be shared genetic risk factors for depression and executive functions deficiency.

3. **Dietary supplementation**
   3.1. Zinc insufficiency **[GENETICS &TREATMENT]**
   a. There is a correlation between depression and zinc insufficiency and this relationship may be linked to certain genetic factors[1].
   b. Zinc insufficiency may be caused by low level of zinc consumption in diet AND/OR impaired zinc absorption caused by medical conditions or other factors. Commonly used medications, such as antacids, diuretics, anticonvulsants, anti-retrovirals, hormones, steroids, and anti-inflammatories, may also impact zinc absorption[1].
   c. Zinc deficiency is associated with weakened immunological functioning, GI issues, ocular and sensory issues[1]. Such problems have also been reported in autism.
   d. Zinc supplementation has been shown to improve mood in subjects with depression, and reduce aggressive behaviors in schizophrenia patients[1].
   e. SHANK3 gene mutations have been reported in autism and Phelan McDermid syndrome (a syndromic form of autism). This gene encodes a protein that is regulated by zinc. Zinc deficiency has been reported in both conditions and zinc supplementation has been proposed as a promising intervention approach[2] (**PMID[1]:** 28713269; **PMID[2]:** 29875651).

Take Home Messages:
-Certain genetic factors may lead to zinc deficiency that has been reported in both depression and autism.
-Zinc supplementation may be considered for treatment of depression or autism.

3.2. Omega-3 insufficiencies [TREATMENT]

a. Omega-3, fatty acids found in fish oil, can modulate key biological pathways, including those involved in inflammation. Omega-3 supplementation has been shown to have therapeutic benefits in the treatment of major depressive disorder and bipolar disorder (**PMID:** 28987035).
b. Omega-3 insufficiencies have been reported in ADHD, depression, schizophrenia, and ASD (**PMID:** 29207548).
c. A meta-analysis study has shown that Omega-3 supplementation cannot be recommended as a single treatment option for behavioral problems in ASD children, but could be used to complement other therapies (**PMID:** 29207548).

Take Home Message:

-Omega-3 supplementation may be considered for treatment of depression in autism.

3.3. Vitamin B deficiencies [GENETICS &TREATMENT]

a. Polymorphisms in genes related to vitamin B metabolism (e.g., MTHFR, MTR) have been associated with depression (**PMID:** 25173634).
b. The level of vitamin B6 may be inversely correlated with circulating folic acid (also known as vitamin B9) (**PMID**: 30390106).
c. Certain polymorphisms in genes involved in folate metabolism (e.g., MTHFR) have been associated with ASD (**PMID:** 23653228).
d. Folinic acid (a metabolically active form of folic acid) improves verbal communication in children with autism and language impairment (**PMID**: 27752075).

Take Home Message:

-Vitamin B supplementation may be considered for treatment of depression in autism.

3.4. Vitamin D level [GENETICS &TREATMENT]

a. Vitamin D plays a role in brain function and behavior (**PMID:** 30214848) and may be associated with depression and anxiety (**PMID:** 30604028; **PMID:** 27750060; **PMID:** 30245372).
b. Genetic variants in several genes (e.g., MAO-A and SERT) have been associated with Vitamin D metabolism (**PMID:** 30008960).

Take Home Message:

-Vitamin D consumption may need to be adjusted for managing depression in autism.

4. Variability in antidepressant response (pharmacogenetics) [GENETICS &TREATMENT]

a. Polymorphisms in certain drug metabolism-related genes have been associated with antidepressant response and may be used to guide treatment in patients (**PMIDs:** 29699889; 30299306), including those with autism (**PMID:** 29398934).

C. Glossary*

- **Comorbidities:** Co-occurring condition
- **Prenatal:** before birth; during or relating to pregnancy
- **Internalizing** (behaviors related to internal feelings, such as anxiety, sadness, feelings of loneliness, negative self-image) and **externalizing** (behaviors that are very obvious and bothersome to others; disruptive; conduct problems, or aggression) **problems.**
- **Executive function:** refers to a set of skills that are required for proper planning, organizing, as well as self-directed behaviors toward achieving a goal and/or making decisions.

D. A potential direction for developing a GO hypothesis

This topic maybe a good candidate to be considered for a GO study because of the following:

- Depression is frequently seen in subjects with autism.
- Some of the underlying genetic risk factors may be shared between autism and depression.
- Dietary supplements of certain nutrients have been suggested as potential treatment options in depression and autism.

Taken together, a combination of genetic risk factors for depression, and history of essential nutrients deficiency may be used to identify ASD subjects who may be at a higher risk for developing depression. Specific intervention therapy, including dietary supplementation of the relevant nutrients, may be used for this ASD subtype to reduce depressive symptoms and improve executive functions.

E. Summary Table

GO Topic: Depression in Autism

	GO Aspects			
**Sub-Topics**	**Genetics**	**Outcomes**	**Treatments**	**Others**
1. Maternal prenatal depression	X	X		
2. Executive functions	X	X		
3. Zinc insufficiency	X		X	
4. Omega 3 polyunsaturated fatty acids insufficiency			X	

GO Hypothesis (summary)

Table summarizes the sub-topics (from literatures and/or suggested by members) that were considered in developing the GO hypothesis-Basic version (Figure).

Table. GO aspects and sub-topics used in the GO hypothesis

**Sub-Topics** [from literatures & members' suggestions]	GO Aspects			
	Genetics	Outcomes	Treatments	Others
1. Maternal prenatal depression	X	X		
2. Executive functions	X	X		
3. Dietary supplementation				
3.1. Zinc insufficiency	X		X	
3.2. Omega 3 insufficiencies			X	
3.3. Vitamin B deficiencies	X		X	
3.4. Vitamin D level	X		X	
4. Pharmacogenetics (variability in antidepressant response)	X		X	

Figure summarizes the developed GO hypothesis as well as potential methods and expected results.

Figure. GO hypothesis

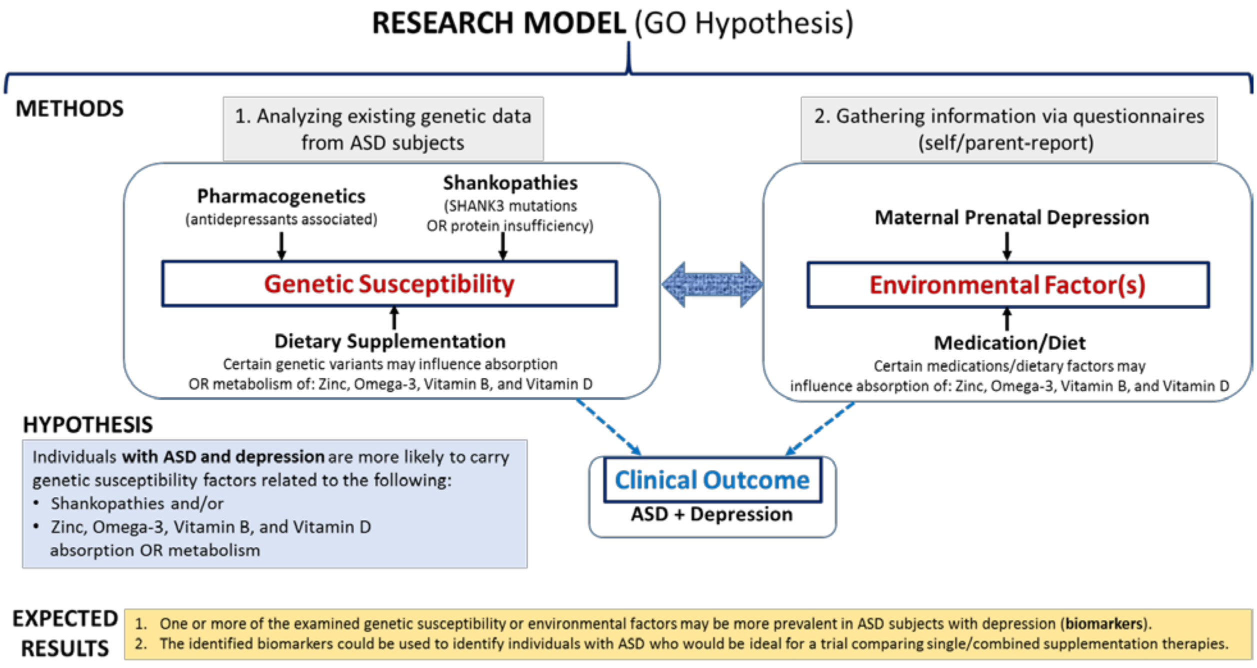

Supplement: Supplementary file 1 — Appendix S1: Supporting Information [file AUR-13-1286-s001.pdf]
